# *Pseudomonas* Species Isolated From *Lotus* Nodules Are Genetically Diverse and Promote Plant Growth

**DOI:** 10.1111/1462-2920.70066

**Published:** 2025-02-27

**Authors:** Yu‐Hsiang Yu, Julian Kurtenbach, Duncan Crosbie, Andreas Brachmann, Macarena Marín Arancibia

**Affiliations:** ^1^ Genetics, Faculty of Biology LMU Munich Planegg‐Martinsried Germany; ^2^ Department of Plant Molecular Biology University of Lausanne Lausanne Switzerland

**Keywords:** *Lotus*, *Mesorhizobium*, plant growth‐promotion, *Pseudomonas* genomic diversity, root nodule symbiosis

## Abstract

Nodules harbour microbial communities composed of rhizobia and other lower‐abundance bacteria. These non‐rhizobial bacteria can promote plant growth. However, their genomic diversity and how this relates to their plant growth‐promoting traits remain poorly investigated. Here, we isolated 14 *Pseudomonas* strains from the nodules of *Lotus* plants, sequenced their genomes, analysed their genomic and phylogenetic diversity, and assessed their ability to promote plant growth. We identified five distinct species, including a novel species named *Pseudomonas monachiensis* sp. nov., with strain PLb12A^T^, as the type strain. Genome analysis of these nodule‐isolated *Pseudomonas* revealed an abundance of genes associated to plant growth‐promoting traits, especially auxin‐related genes, compared to closely related type strains. In accordance, most nodule‐isolated *Pseudomonas* strains enhanced shoot growth of *Lotus burttii*, while only some promoted root growth or early onset of root hair proliferation. However, none of the strains significantly affected the ability to form nodules. Overall, our findings highlight the genotypic diversity and the plant growth‐promoting potential of nodule‐isolated *Pseudomonas* and underscore their possible applications in mixed inocula with rhizobia.

## Graphical abstract

*Pseudomonas* co‐inhabit the root nodules of *Lotus* plants alongside *Mesorhizobium*. Our phylogenomic analysis reveals high genomic diversity among these *Pseudomonas* strains and widespread plant growth‐promoting abilities. This diversity suggests a potential for varied mechanisms to enhance plant health and potentially symbiotic efficiency.

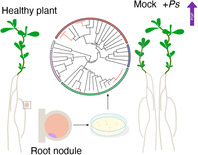

## 1 Introduction

Leguminous plants form a mutualistic symbiosis with nitrogen‐fixing rhizobia, which they house within specialised cells of root organs called nodules (Venado et al. 2020). This symbiosis facilitates nitrogen fixation, converting inert atmospheric nitrogen into ammonia, a crucial nutrient for plant growth, thereby enabling plants to thrive in nitrogen‐poor soils and enhancing soil fertility (Masson‐Boivin and Sachs 2018).

In recent years, microbiome studies have uncovered the presence of non‐rhizobial bacteria inside nodules (Martínez‐Hidalgo and Hirsch 2017; Velázquez et al. 2017; Mayhood and Mirza 2021). Nodule communities are composed by a variety of non‐rhizobial genera, including but not limited to *Bacillus*, *Enterobacter*, *Flavobacterium*, *Pseudomonas*, *Sphingomonas* and *Streptomyces* (Hnini and Aurag 2024; Yu, Crosbie, and Marín Arancibia 2025). These non‐rhizobial bacteria can influence plant growth, nodulation and overall health. For instance, certain non‐rhizobial bacteria help plants tolerate stresses such as salinity (Egamberdieva et al. 2017; Noori et al. 2018), drought (Noori et al. 2018), heavy metals (Chiboub et al. 2016; Abdelkrim et al. 2020; Flores‐Duarte et al. 2022) and pathogens (Egamberdieva et al. 2017; Tokgöz et al. 2020; Dhole and Shelat 2022), and thus have great agronomical potential.

Among the non‐rhizobial bacteria inhabiting root nodules, members of the genus *Pseudomonas* are frequently detected in nodules spanning diverse legume crops including *Glycine max* , *Lens culinaris* , *Phaseolus vulgaris* and *Vigna radiata* (Hnini and Aurag 2024; Yu, Crosbie, and Marín Arancibia 2025). However, the exact roles of *Pseudomonas* in these systems remain unexplored. Many studies have focused on the abilities of nodule‐isolated *Pseudomonas* strains to promote plant growth or in vitro plant growth‐promoting traits (PGPT). However, the causal link between in planta and in vitro phenotypes is often missing. Moreover, the genomes of nodule‐isolated *Pseudomonas* are rarely sequenced, which has hindered the study of their genomic diversity and in silico predictions of the potential functions of these *Pseudomonas*.

In this study, we aimed to elucidate the genomic and phenotypic diversity of *Pseudomonas* colonising *Lotus* nodules. To this end, we isolated bacteria from nodules of three *Lotus* species used as trap plants, we sequenced and analysed the genomes of 14 isolates, and characterised a novel species designated as *Pseudomonas monachiensis* sp. nov. Additionally, we evaluated the plant growth promotion potential of these *Pseudomonas* strains in silico, in vitro and in planta, and their influence on the root nodule symbiosis between *Lotus burttii* and *Mesorhizobia* bacteria.

## 2 Materials and Methods

### 2.1 Soil Collection and Plant Inoculation

Soil samples were collected from two sites (site 1: 48°06′29.9″N, 11°27′38.9″ E, and site 2: 48°06′33.2″N, 11°27′41.4″ E) in Munich, Germany. Seeds of *Lotus burttii* B‐303 (Seed bag 91,105), *Lotus japonicus* Gifu B‐129 (Seed bag 110,913) and *Lotus corniculatus* cv. Leo (Andreae Saaten, Regensburg, Germany) were scarified, surface sterilised with a sterilising solution (1.2% NaOCl, 1% SDS), and germinated in 0.5× B5 agar medium (Gamborg et al. 1968) for 3 days in dark followed by 3 days in a long‐day photoperiod (16 h light, 8 h dark) at 24°C as described previously (Crosbie et al. 2022). Seedlings were transferred to tulip shaped jars (J. WECK Company, Germany) filled with a 1:2 mixture of sterile sand:vermiculite supplemented with 40 mL FAB medium (Gong et al. 2022) containing 0.1 mM KNO_3_ and recovered for 3 days in a long‐day photoperiod at 24°C (Liang et al. 2019). Seedlings were inoculated with soil suspensions prepared as described before (Crosbie et al. 2022). Plants were grown under a long‐day photoperiod at 24°C for 5 weeks before harvesting and phenotyping. Pink nodules from healthy plants were collected and used for bacterial isolation (Crosbie et al. 2022).

### 2.2 Isolation of Bacteria

Nodules were surface sterilised with 70% ethanol for 1 min followed by 2% NaOCl for 2.5 min, and extensive water washing, as described previously (Crosbie et al. 2022). Surface sterilised nodules were crushed and spread onto various agar media, including 20Q medium supplemented with 3.8% w/v mannitol (Werner et al. 1975), Luria‐Bertani (LB) medium (Bertani 1951), yeast mannitol medium (Vincent 1970), *Pseudomonas* minimal medium (Sandman and Ecker 2014) and tryptone soy medium (Gould et al. 1985). The cultures were then incubated at 28°C until colonies emerged. Single colonies were isolated 7–9 times until pure cultures were attained.

### 2.3 Bacteria Strains and Growth Conditions

Strains used in this work are listed in Table S1. *Pseudomonas* strains were cultivated in LB medium at 28°C and 180 rpm for 16 h. *Mesorhizobium* sp. DC‐1.5 was cultivated in 20Q medium at 28°C and 180 rpm for 48 h.

### 2.4 DNA Extraction, Whole Genome Sequencing, Assembly and Annotation

The genomic DNA of 14 nodule‐isolated *Pseudomonas* strains were extracted using the CTAB method (William et al. 2012). The concentration of the DNA samples was checked by Nanodrop ND‐1000 (Thermo Fisher Scientific, USA) and Qubit 2.0 fluorometer (Invitrogen, USA). Genomic sequencing libraries were constructed from 1 ng of genomic DNA with the Nextera XT DNA Sample Preparation Kit (Illumina, USA) according to the manufacturer's protocol. The library was quality controlled by analysis on an Agilent 2000 Bioanalyzer with the Agilent High Sensitivity DNA Kit (Agilent Technologies, USA) for fragment sizes of ca. 400–900 bp. Sequencing on a MiSeq sequencer (Illumina; 2 × 300 bp paired‐end sequencing, v3 chemistry) was performed in the Genomics Service Unit (LMU Biocenter, Martinsried, Germany). The raw reads were controlled for quality using FASTQC version 0.11.9 with default settings (Andrews 2010). Adapters and low‐quality bases were trimmed using Trimmed Galore version 0.6.7 selecting “nextera” as the adapter sequence (Krueger 2012) and controlled for quality after trimming using FASTQC version 0.11.9 with default settings (Andrews 2010). A de novo genome assembly was performed using Shovill version 1.1.0 selecting “Spades” as assembler (Seemann 2017). The quality of the assembly was evaluated using QUAST version 5.2.0 with default settings for assessing the genome size, contig number, N50, L50, GC content and cumulative length (Mikheenko et al. 2018).

For long read sequencing, the genomic DNA of strain PLb12A^T^ was isolated using the NucleoBond HMW DNA kit (MACHEREY‐NAGEL GmbH, Germany), following the manufacturer's protocol. Long‐read sequencing using a PromethION (ONT—Oxford Nanopore Technologies) was performed by Microsynth AG (Balgach, Switzerland). The ONT raw reads were trimmed using Filtlong version 0.2.1, with the minimum read length set to 1000 bp (Wick 2017). The quality of the trimmed reads was assessed using FastQC version 0.12.1 under default settings (Andrews 2010). Additionally, the read quality before and after trimming was evaluated using NanoPlot version 1.43.0, also under default settings (De Coster 2018).

The trimmed ONT reads were assembled using Flye version 2.9.5 with the “–nano‐corr” parameter enabled for Nanopore‐corrected reads (Kolmogorov et al. 2019). The resulting assembly was polished in two stages. For the first polishing, Medaka version 1.7.2 was used with the model selected based on the sequencing data (Oxford Nanopore Technologies 2021). For the second polishing step, raw Illumina MiSeq reads were processed by trimming with Fastp version 0.23.4, using the following parameters: required length ≥ 30 bp, quality trimming at both the 5′ and 3′ ends, window size = 4 bp, and mean quality threshold ≥ 20 (Chen et al. 2018). Polishing was then performed using Polypolish version 0.5.0 under default settings (Wick and Holt 2022) and Pilon version 1.20 with the “–changes” file option enabled (Walker et al. 2014).

The quality of the final assembly was evaluated using QUAST version 5.2.0 (Mikheenko et al. 2018). Additionally, Snippy version 4.6.0 was employed with default settings to identify variants between the Illumina MiSeq and ONT reads (Seemann 2015).

The genomes were annotated using Prokka version 1.14.6 and the integrated *Pseudomonas* genus‐specific BLAST database. Contigs smaller than 200 bp were not used in the annotation process (Seemann 2014). The genome completeness was evaluated using Benchmarking Universal Single‐Copy Orthologs (BUSCO) version 5.4.6 with default settings. A value exceeding 90% was considered indicative of a well‐assembled and annotated genome, as described previously (Simão et al. 2015). Additionally, the presence of the 16S rRNA gene sequence was verified using ContEst16S with default settings for all isolates (Lee et al. 2017), and by Sanger sequencing using primers 27F and 1492R (Lane 1991) for *P. monachiensis* isolates.

All bioinformatic tools used in this section, except ContEst16S, were accessed via the Galaxy Server and the Galaxy Europe Server (The Galaxy Community 2024).

### 2.5 Phylogenetic Analyses and Core Genome Analysis

Phylogenetic analyses were conducted for 16S rRNA gene, core genome and whole genome sequences. The 16S rRNA gene and whole genome phylogenetic trees were constructed by uploading the assembled genomes to the Type Strain Genome Server (TYGS), a widely utilised tool for phylogeny analysis and species identification (Meier‐Kolthoff and Göker 2019; Lalucat et al. 2020). Additionally, the assembled and polished complete genome of strain PLb12A^T^ was further analysed using GTDB‐Tk version 2.4.0 under default settings (Chaumeil et al. 2022).

The phylogenetic trees were generated using FastME version 2.1.6.1 through the Genome BLAST Distance Phylogeny (GBDP) approach. The GBDP distances were computed based on both 16S rRNA gene and whole genome sequences, with branch lengths scaled according to the GBDP distance formula *d5* (Meier‐Kolthoff et al. 2013; Lefort et al. 2015).

For the core genome phylogenomic tree, type strains were selected based on the whole genome phylogeny constructed using TYGS and a recent study on *Pseudomonas* taxonomy, which introduces new species closely related to the subgroup of this study, offering enhanced resolution for identification (Girard et al. 2021). The annotated genomes of 14 nodule‐isolated *Pseudomonas* strains, and 36 other *Pseudomonas* strains were compared using Roary version 3.13.0 with an amino acid identity threshold of 70% (Page et al. 2015; Sawada et al. 2022). The core genome alignment was performed using MAFFT version 7.508 with default settings (Katoh and Standley 2013). Subsequently, the core genome phylogenomic tree was constructed using RAxML‐HPC2 version 8.2.12 with 1000 replicates of bootstrap calculation (Nguyen et al. 2015). Strains used in the phylogenetic analyses were listed in Table S2. All trees were visualised in iTOL version 6.8 (Letunic and Bork 2021).

Number of core and cloud genes in nodule‐isolated *Pseudomonas* and their closest type strains was estimated using Roary version 3.13.0 with a 90% amino acid identity threshold (genus boundaries). Flower and upset plots were visualised in R version 4.2.2 (R Core Team 2013), using the Plotrix package (Lemon 2006) and UpSetR package (Conway et al. 2017), respectively.

### 2.6 Average Nucleotide Identity (ANI) and Digital DNA–DNA Hybridisation (dDDH) Analysis

ANI was calculated using FastANI and OrthoANI. ANI values were computed for the genomes of nodule‐isolated *Pseudomonas* strains in comparison to closely related type strains based on the phylogeny analyses in TYGS using default settings in FastANI version 1.3 (Jain et al. 2018) and OrthoANI version 0.93.1 (Lee et al. 2016). dDDH values were determined using the TYGS server with default settings and the formula *d4* (Auch et al. 2010; Meier‐Kolthoff and Göker 2019).

### 2.7 Phenotypic Characterisation of *Pseudomonas* Isolates for Taxonomic Classification

Phenotypic characterisation was conducted for *P. monachiensis* isolates and type strains of closely related species. The colony morphology of isolates was determined after 24 h of incubation on LB agar at 28°C. The plate overview and close‐up pictures of the colonies were captured using a DSC‐HX400V camera (Sony, Japan). Individual colonies were imaged with a M165 FC stereomicroscope (Leica, Germany).

The ability of strains to grow at different temperatures, their pH tolerance, antibiotic resistance and salt tolerance was systematically characterised. For all assays bacteria were grown in LB broth. Two independent experiments were conducted, each comprising three technical replicates.

To determine the range of temperatures in which isolates can grow, 50 μL of bacterial suspensions with an OD_600_ of 1.0 were spread onto LB agar medium and incubated at 4°C, 24°C, 28°C or 37°C. Growth was observed over a 3‐day period and recorded as strong (+), weak (+^w^) or no growth (−).

To determine pH tolerance, bacteria cultures were diluted to an OD_600_ of 0.1 and grown in LB broth with pH values ranging from 5 to 10 on 48‐well plates. Cultures were incubated at 28°C and 180 rpm for 24 h.

For antibiotic resistance assays, bacterial suspensions with an OD_600_ of 1.0 were spread onto LB agar. Filter papers containing 100 μg/mL ampicillin, 100 μg/mL carbenicillin, 50 μg/mL fosfomycin, 15 μg/mL gentamycin, 50 μg/mL kanamycin, 50 μg/mL neomycin, 100 μg/mL spectinomycin, 100 μg/mL streptomycin and 10 μg/mL tetracycline, were placed onto the bacterial spread. Plates were incubated at 28°C for 1 day and results were recorded as resistance (R), weak susceptibility (S^w^) or strong susceptibility (S^+^).

For salt tolerance assays, bacterial suspensions with an OD_600_ of 1.0 were spread onto LB agar medium supplemented with NaCl at varying final concentrations (1%, 2%, 3%, 4%, 4.5% and 5% w/v). Plates were incubated at 28°C for 7 days and results were recorded as strong (+), weak (+^w^) or no growth (−).

To determine the fatty acid composition, bacteria were grown in LB broth at 28°C and 180 rpm for 16 h. Cells were collected and freeze‐dried using an Alpha 1–2 LD plus lyophiliser (Martin Christ GmbH, Germany). The whole‐cell fatty acids analysis was conducted by the Leibniz Institute DSMZ (DSMZ) based on the GC–MS 7000D system (Agilent Technologies, USA), as described previously (Sasser 1990).

### 2.8 Genome Analysis of the Nodule‐Isolated *Pseudomonas* Species

The genomes of the nodule‐isolated *Pseudomonas* were analysed using the CGView (Proksee) server (Grant et al. 2023). The bacterial mobile genetic elements (MGEs), putative horizontal gene transfer events and antibiotic resistance genes were predicted by the mobileOG‐db database version 1.1.3 (Brown et al. 2022), Alien Hunter version 1.1.0 (Vernikos and Parkhill 2006), and CARD RGI version 1.2.0, respectively, using default settings (Alcock et al. 2023).

### 2.9 In Silico Prediction of PGPT

Bacterial PGPT were annotated using the blastp + hmmer (strict mode) mapping against the PGPT Ontology, within the PGPT‐Pred tool provided by the PLaBAse server (Patz et al. 2021; Ashrafi et al. 2022). Annotated genomes of 14 nodule‐isolated *Pseudomonas* strains, 19 closely related strains, and 29 PGP *Pseudomonas* strains described in previous studies were analysed using default settings (Table S2). The PGPT‐Pred algorithm generated six hierarchical levels of traits, ranging from general categories to specific genes. Within Level 1, distinctions are made between direct and indirect effects, while at Level 2, the algorithm generates eight sub‐categories including biofertilisation, bioremediation, phytohormone/plant signal production, colonisation, competitive/exclusion, plant immune response/stimulation, stress control/biocontrol and putative functions. The sub‐category of phytohormone/plant signal production was further explored from Levels 3 to 6. The number of total traits and traits in Levels 2 and 5 were represented as a heat map with *z*‐score calculation in R version 4.2.2 (R Core Team 2013), using the Pheatmap package (Kolde 2019). The cladogram was constructed based on the *rpoD* gene phylogeny. *rpoD* was extracted from the genomes and aligned using MAFFT version 7.526 (Katoh and Standley 2013). The resulting guide tree was visualised with iTOL version 6.8 (Letunic and Bork 2021). IAA‐related genes (Level 6) were extracted from the annotated genomes and their KEGG ID was obtained using PGPT Ontology. To reconstruct putative IAA biosynthetic pathways, the annotated genomes were uploaded to BlastKOALA and KofamKOALA in the KEGG database (Kanehisa and Goto 2000). After pathway reconstruction, individual gene sequences were manually blasted to confirm their presence in the genomes of the tested strains.

### 2.10 In Vitro PGP Assays

Siderophore production and phosphate solubilisation were determined using chrome azurol S (CAS) agar (Alexander and Zuberer 1991) and Pikovskaya's agar (Pikovskaya 1948), respectively. Suspensions of nodule‐isolated *Pseudomonas* strains were adjusted to an OD_600_ of 1.0 using sterile water and three replicates of each strain were spotted onto the respective agar plates. The plates were incubated at 28°C for 24 h. Measurements were taken every 24 h for 7 days to monitor growth and the formation of halo zones. Two independent experiments were performed for each assay.

In addition, strains were characterised for their nitrogen reduction, indole production, carbon assimilation and other enzymatic activities using the API 20 NE system, following the manufacturer's protocol.

### 2.11 Plant Growth Assays

In planta assays were conducted to evaluate the PGP capabilities of nodule‐isolated *Pseudomonas* strains under axenic conditions (Table S1). Seeds of *L. burttii* (Seed bag 92,876) were scarified, surface sterilised and germinated in 0.5× B5 agar medium at 24°C for 3 days in dark followed by 3 days under a long‐day photoperiod as described in previous section.

6 day‐old seedlings were transferred to tulip‐shaped jars (J. WECK Company, Germany) filled with a sterile 1:2 mixture of sand:vermiculite supplemented with 40 mL FAB medium containing 5 mM KNO_3_ (FAB_5mM_) as the nitrogen source, then recovered at 24°C under a long‐day photoperiod for 3 days. *Pseudomonas* suspensions were adjusted to an OD_600_ of 0.005 with FAB_5mM_, and each seedling was inoculated with 1 mL of the respective *Pseudomonas* suspension. Control seedlings were treated with 1 mL of FAB_5mM_. The plants were grown at 24°C under a long‐day photoperiod. Each treatment comprised 20 plants, and two independent experiments were conducted. 4 weeks after inoculation, the plants were harvested and phenotypic traits, including shoot and root length, as well as root fresh weight, were assessed.

To assess the influence of nodule‐isolated *Pseudomonas* strains on root development, seedlings with comparable root length were transplanted onto fresh 0.5× B5 agar medium and incubated at 24°C for two additional days under a long‐day photoperiod. *Pseudomonas* suspensions were washed and adjusted to an OD_600_ of 0.005 with sterile water and 20 μL spots were applied to the root tips of each plant, while 20 μL of sterile water served as mock. Droplets were left to dry before vertically growing the treated plants at 24°C for 5 days under a long‐day photoperiod. Each treatment comprised 20 plants, and two independent experiments were conducted. At 5 days post inoculation, root phenotypes were examined using a MF165 FC stereomicroscope (Leica, Germany).

### 2.12 Nodulation Assays

For co‐inoculation of nodule‐isolated *Pseudomonas* with *Mesorhizobium* sp. DC‐1.5, seedlings were transferred to tulip shaped jars (J. WECK Company, Germany) containing a sterile 1:2 mixture of sand:vermiculite supplemented with 40 mL FAB medium containing 0.1 mM KNO_3_ (FAB_0.1mM_) as nitrogen source. Both *Pseudomonas* and *Mesorhizobium* sp. DC‐1.5 suspensions were diluted to an OD_600_ of 0.01 using FAB_0.1mM_. Subsequently, they were mixed in a 1:1 ratio to achieve a final OD_600_ of 0.005 for both *Pseudomonas* strains and *Mesorhizobium* sp. DC‐1.5. One millilitre of this mixture was then inoculated onto each seedling. Single inoculation of *Mesorhizobium* sp. DC‐1.5 was used as control. The plants were grown for 3 weeks at 24°C under a long‐day photoperiod, after which plants were harvested and the number of nodules was counted. Each treatment comprised 20 plants, and two independent experiments were conducted.

### 2.13 Statistical Analyses

One‐way ANOVA followed by Tukey's HSD test was performed to evaluate differences among in vitro phenotypes using R version 4.2.2 (R Core Team 2013). Statistical analyses of plant growth assays were conducted using Student's *t*‐tests in R version 4.2.2 (R Core Team 2013).

## 3 Results

### 3.1 Isolation and Genomic Characterisation of Nodule‐Isolated *Pseudomonas* Strains

To obtain endophytic *Pseudomonas* inhabiting *Lotus* nodules, we used *L. burttii*, *L. japonicus* and *L. corniculatus* plants as traps. We inoculated them with soil suspensions extracted from two soils as described previously (Crosbie et al. 2022), and isolated bacteria from surface sterilised nodules. *Pseudomonas* were recovered exclusively from pink coloured nodules of green and healthy‐looking plants. 13 isolates were obtained from *L. burttii*, while one isolate was recovered from a pink *L. corniculatus* nodule (Table S1).

To characterise the nodule‐isolated *Pseudomonas*, the genomes of 14 isolates were sequenced, assembled and annotated (Table S3). The genome size, GC content, BUSCO values, number of coding sequences and RNA counts were listed in Table S3.

### 3.2 Nodule‐Isolated *Pseudomonas* Belong to Five Distinct Species

To determine the relationship among the isolates, we conducted phylogenetic analyses, including type strains of the species closest to the nodule‐isolated strains. First, a phylogenetic tree was constructed based on the 16S rRNA gene on the TYGS server, which assigned the 14 nodule‐isolated *Pseudomonas* to the *Pseudomonas fluorescens* group (Figure S1). To achieve higher resolution, we conducted a core genome phylogenomic analysis using the core genome (1423 genes) predicted from a comparison of 50 strains (Table S4). The nodule isolates were classified into five clusters and distributed across three subgroups within the *P. fluorescens* group: two clusters in the *Pseudomonas jessenii* subgroup, two clusters in the *Pseudomonas corrugata* subgroup, and one in the *P. fluorescens* subgroup (Figure S2).

**FIGURE 1:**
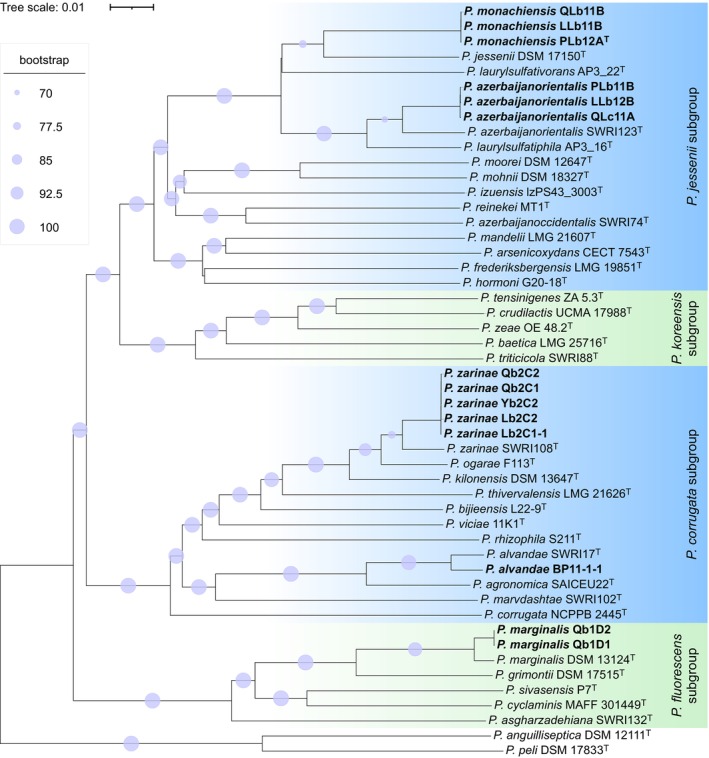
Whole genome phylogenetic tree of nodule‐isolated *Pseudomonas*. The phylogenetic tree was constructed using the genomes of 14 nodule‐isolated *Pseudomonas* and 36 *Pseudomonas* type strains in the TYGS server (Meier‐Kolthoff and Göker 2019). Two groups were included in the tree: *Pseudomonas fluorescens* group (including *Pseudomonas jessenii* , *Pseudomonas koreensis* , *Pseudomonas corrugata* , and *P. fluorescens* subgroups) and the *Pseudomonas anguilliseptica* group. The type strains *P. anguilliseptica* DSM 12111^T^ and *Pseudomonas peli* DSM 17833^T^ in the *P. anguilliseptica* group were used as outgroup. Nodule isolates are highlighted in bold. Branches are annotated with pseudo‐bootstrap support values (> 70%) from 100 replications, averaging at 84.5% branch support.

For the precise species classification, a whole genome phylogenetic tree was generated using the TYGS server. Nodule isolates clustered together with the type strains of described *Pseudomonas* species, namely strains LLb11B, PLb12A^T^ and QLb11B with *P. jessenii* , strains LLb12B, PLb11B and QLc11A with *Pseudomonas azerbaijanorientalis*, strains Lb2C1–1, Lb2C2, Qb2C1, Qb2C2 and Yb2C2 with *Pseudomonas zarinae*, strain BP11‐1‐1 with *Pseudomonas alvandae*, and strains Qb1D1 and Qb1D2 with *Pseudomonas marginalis* (Figure 1).

To determine if any of the isolates belonged to a novel *Pseudomonas* species, we conducted ANI analyses. ANI values of 95% are accepted as a species definition threshold (Richter and Rosselló‐Móra 2009). First, we conducted a FastANI analysis utilising genome sequences from a previous taxonomic study (Girard et al. 2021). Most isolates had > 97% similar to type strains (Table S5). The only exception were isolates LLb11B, PLb12A^T^ and QLb11B, which exhibited a similarity of 94.1% to the type strain *P. jessenii* DSM 17150^T^ and 92.2% to the type strain *Pseudomonas laurylsulfativorans* AP3_22^T^ (Table S5). For a more robust analysis of strains LLb11B, PLb12A^T^ and QLb11B we used OrthoANI, which estimates ANI using orthologous fragments (Lee et al. 2016; Jain et al. 2018) and dDDH (Auch et al. 2010). The threshold for defining bacterial species based on dDDH is set at 70% (Stackebrandt and Goebel 1994). OrthoANI results revealed that the three isolates shared a 93.8%–93.9% similarity with *P. jessenii* DSM 17150^T^ and a 92% similarity with *P. laurylsulfativorans* AP3_22^T^ (Table S6). Regarding dDDH, the three isolates shared 100% similarity with each other, but only 54.2% similarity when compared to *P. jessenii* DSM 17150^T^ and 45.8% when compared to *P. laurylsulfativorans* AP3_22^T^ (Table S7). In summary, isolates LLb11B, PLb12A^T^ and QLb11B shared an OrthoANI value below 95% (Richter and Rosselló‐Móra 2009), and a dDDH value below 70% (Stackebrandt and Goebel 1994) compared to the closest described type strains, suggesting that they belong to a novel species within the genus *Pseudomonas*. For greater precision we sequenced the complete genome of strain PLb12A^T^ using ONT. This genome was analysed using the GTDB‐Tk database to confirm species assignment (Chaumeil et al. 2022). The results revealed that the closest type strain is *P. jessenii* DSM 17150^T^, with an ANI value of 94.01% (Table S8).

To support these conclusions, we conducted a thorough phenotypic characterisation of these isolates and compared them to the type strains of the closest described species *P. jessenii* DSM 17150^T^ and *P. laurylsulfativorans* AP3_22^T^. When grown on LB agar medium, LLb11B, PLb12A^T^ and QLb11B developed colonies that were circular, convex, smooth, opaque and beige to slightly yellowish in colour (Figure S3). The usual diameter of a single colony after 24 h of growth at 28°C was between 1.7 and 2.2 mm (Figure S3). All strains exhibited robust growth within a temperature range of 24°C–28°C, with slower growth observed at 37°C. They also grew across a broad pH range from 5 to 9. Notably, all strains belonging to the potential new species exhibited growth on media supplemented with up to 5% NaCl, whereas *P. jessenii* DSM 17150^T^ and *P. laurylsulfativorans* AP3_22^T^ displayed growth only up to 3% and 4% NaCl, respectively (Table S9). All three *P. monachiensis* isolates and *P. laurylsulfativorans* AP3_22ᵀ exhibited strong sensitivity to fosfomycin and mild sensitivity to kanamycin. In contrast, *P. jessenii* DSM 17150^T^ showed mild sensitivity to both fosfomycin and kanamycin. All strains were able to grow in the presence of the other tested antibiotics (Table S9).

Fatty acid composition varies significantly between different bacterial species and is frequently used in bacterial taxonomy for species identification (Tindall et al. 2010). The major fatty acids of LLb11B, PLb12A^T^ and QLb11B were determined using a GC–MS 7000D and compared to the profiles of *P. jessenii* DSM 17150^T^, and *P. laurylsulfativorans* AP3_22^T^ as reported in (Furmanczyk et al. 2018). Strains LLb11B, PLb12A^T^ and QLb11B contained fatty acids, C_10:0 3‐OH_, C_12:0 3‐OH_, C_12:1 3‐OH ω7c_, C_16:0_ and C_17:0 cyclo ω7c_ as major constituents, consistently detected across all three strains (Table S9). Additionally, trace amounts of unidentified fatty acids were noted in each strain (Table S9).

The strains were characterised for their nitrogen reduction, indole production, carbon assimilation and various enzymatic activities using the API 20 NE system. Strains LLb11B, PLb12A^T^ and QLb11B demonstrated β‐glucosidase activity and were capable of assimilating all tested carbon sources and acids, with the exceptions of D‐maltose and adipic acid (Table S10).

Based on these results, we posit that isolates LLb11B, PLb12A^T^ and QLb11B belong to a novel *Pseudomonas* species, for which we propose the name, *P. monachiensis* sp. nov. with strain PLb12A^T^ designated as the type strain (DSM 116615^T^ = CCOS 2100^T^ = LMG 33494^T^).

### 3.3 *Pseudomonas* Isolated From *Lotus* Nodules Are Genomically Diverse

**FIGURE 2:**
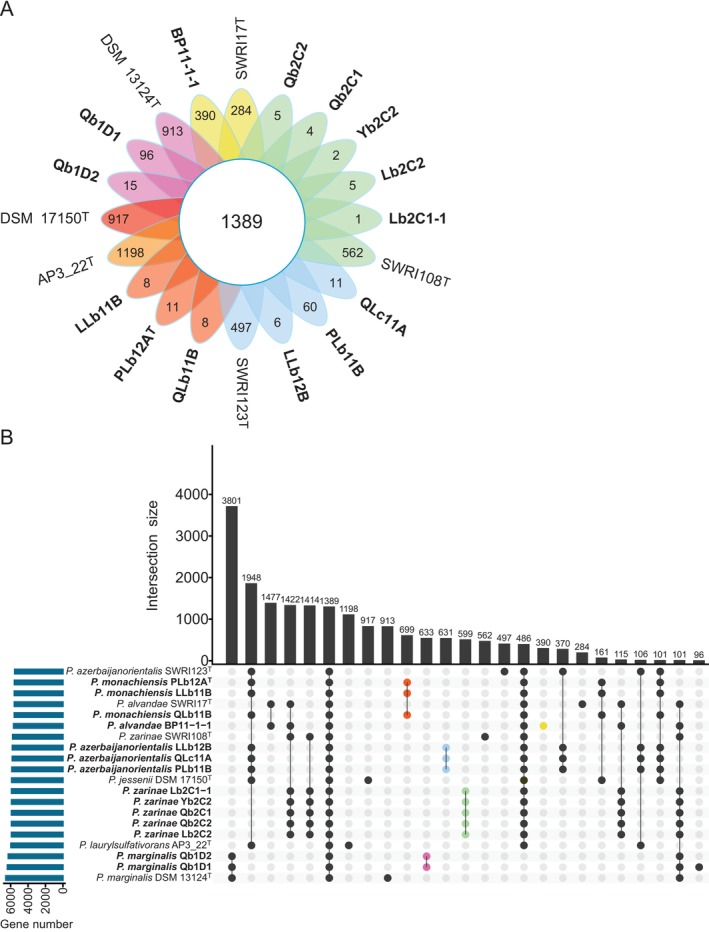
Core genome analysis of 14 nodule‐isolated *Pseudomonas* strains and their closest type strains. Estimation of conserved and strain‐specific genes was conducted using Roary version 3.13.0 (Page et al. 2015) with a 90% amino acid identity threshold. The Flower (A) and UpSet (B) plots were created in R version 4.2.2 (R Core Team, 2013), using the Plotrix (Lemon 2006) and the UpSetR package (Conway et al. 2017), respectively. Nodule isolates are highlighted in bold. (A) Flower plot depiction of the number of core genes (central circle) and of strain‐specific genes (flower petals). *Pseudomonas alvandae* (SWRI17^T^ and BP11‐1‐1); *Pseudomonas marginalis* (DSM 13124^T^, Qb1D1, and Qb1D2); *Pseudomonas jessenii* (DSM 17150^T^); *Pseudomonas laurylsulfativorans* (AP3_22^T^); *Pseudomonas monachiensis* (PLb12A^T^, LLb11B, and QLb11B); *Pseudomonas azerbaijanorientalis* (SWRI123^T^, LLb12B, PLb11B, and QLc11A); and *Pseudomonas zarinae* (SWRI108^T^, Lb2C1–1, Lb2C2, Qb2C1, Qb2C2, and Yb2C2). (B) UpSet plot depicting the number of genes in the top 25 intersections. Coloured dots highlight the intersections representing the nodule specific species.

To visualise the genomic diversity of nodule‐isolated *Pseudomonas*, we compared their annotated genomes to the genomes of the closest type strains. We estimated the core annotated genome using a 90% amino acid identity threshold. The analysis revealed that 1389 genes were shared between the nodule‐isolated *Pseudomonas* and their closest type strains (Figure 2A). For nodule‐isolated strains, there were up to 699 genes specific to *P. monachiensis*, 633 genes specific to *P. marginalis* , 631 genes specific to *P. azerbaijanorientalis*, 599 genes specific to *P. zarinae* and 390 genes specific to *P. alvandae* BP11‐1‐1 (Figure 2B). All genes shared among nodule‐isolated strains are also shared with closely related type strains, with none unique to all nodule isolates.

As symbiotic and virulence factors are often located in MGEs, we assessed the presence of putative bacterial MGEs, potential horizontal gene transfer events and antibiotic resistance genes in the genomes of nodule‐isolated *Pseudomonas* strains. Using the mobileOG‐db database, we predicted traits, including stability, transfer, defence mechanisms, interorganism transfer events, phage‐related traits, replication, recombination, nucleic acid repair and integration/excision events, yielding from 125 to 208 total events (Table S3). Horizontal gene transfer events, as predicted by Alien Hunter, ranged from 114 to 200, while antibiotic resistance genes, identified by the CARD RGI, ranged from 5 to 7 (Table S3). Variations in the frequency of horizontal gene transfer events were observed, even within the same species, underscoring their genomic diversity. Symbiotic genes, such as genes involved in the synthesis of nodulation factors (*nodABCD*) or genes encoding for the nitrogenase enzyme (*nifHDK*) were absent from all genomes.

### 3.4 Nodule‐Isolated *Pseudomonas* Strains Are Enriched in PGP Genes and Exhibit PGP Traits In Vitro

**FIGURE 3:**
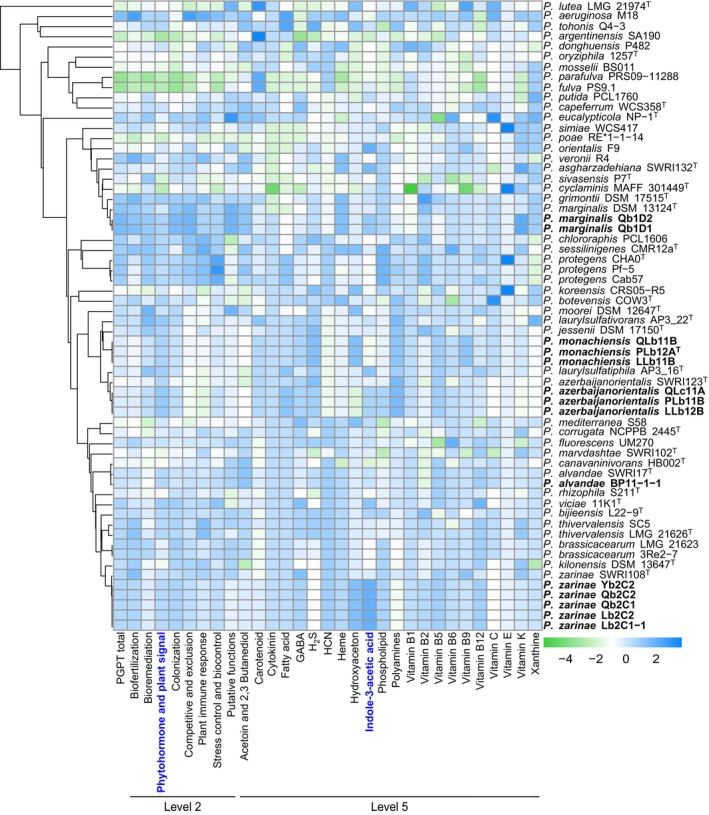
Heat map representation of plant growth‐promoting traits functional annotation. Annotated genomes of 14 nodule isolates, 29 characterised plant growth‐promoting *Pseudomonas* strains, and 19 closely related strains were uploaded to PGPT‐Pred in PLaBAse server. The annotation of bacterial plant growth‐promoting traits was performed using the blastp + hmmer (strict mode) mapping against the PGPT Ontology. Eight groups at Level 2, and 22 subcategories within the phytohormone and plant signal group at Level 5 were used for heat map construction in R version 4.2.2 (R Core Team, 2013), using the Pheatmap package (Kolde 2019). Clustering for the heatmap was based on the *rpoD* gene. The *rpoD* gene was extracted from the genomes and aligned using MAFFT version 7.526 (Katoh and Standley 2013), and the resulting guide tree was visualised with iTOL version 6.8 (Letunic and Bork 2021). The phytohormone and plant signal group (Level 2), as well as the indole‐3‐acetic acid subcategory (Level 5) are highlighted in bold blue. Nodule isolates are indicated in bold. The bluish and greenish colours depict enriched and depleted plant growth‐promoting traits, respectively based on a *z*‐score.

Although often lacking symbiotic genes, *Pseudomonas* isolated from legume nodules frequently have PGP abilities (Hnini and Aurag 2024; Yu, Crosbie, and Marín Arancibia 2025). To assess the potential PGP activities of all nodule isolates, functional annotation was conducted using PGPT‐Pred in PLaBAse server (Patz et al. 2021) and compared them to strains with known PGP activities, such as *Pseudomonas protegens* CHA0^T^ (Jousset et al. 2014), *Pseudomonas simiae* WCS417 (Pieterse et al. 2021), and *P. fluorescens* UM270 (Hernández‐Salmerón et al. 2017). PGPT‐Pred categorises traits into eight main functional groups: biofertilisation, bioremediation, phytohormone/plant signal production, colonisation, competitive/exclusion, plant immune response/stimulation, stress control/biocontrol and other putative functions. The genomes of most nodule‐isolated *Pseudomonas* strains were enriched in various functional groups compared to their respective type strains (Figure 3 and Table S11). Notably, nodule isolates of *P. zarinae*, *P. azerbaijanorientalis* and *P. marginalis* showed enrichment in genes within the phytohormone/plant signal production category. All these isolates were enriched in IAA biosynthesis genes; additionally, *P. zarinae* isolates showed abundance in genes for hydroxyaceton and vitamins B2 and B12, *P. azerbaijanorientalis* isolates were enriched in genes for vitamins B2 and B6, and *P. marginalis* isolates exhibited enrichment in genes for vitamin K. Furthermore, *P. monachiensis* nodule isolates displayed more genes for IAA and vitamin B9 compared to *P. jessenii* DSM 17150^T^ (Figure 3, Table S11).

To determine if the nodule‐isolated *Pseudomonas* strains possessed complete pathways necessary to synthesise IAA from tryptophan, we reconstructed the main five IAA biosynthetic pathways of nodule‐isolated *Pseudomonas* and their closest type strains (Figure S4, Table S12). To this end, we mapped trait identifiers from the PLaBAse database to the KEGG database and manually searched the NCBI database using blast (Johnson et al. 2008). Among the nodule‐isolated strains, only *P. alvandae* BP11‐1‐1, *P. monachiensis* LLb11B, PLb12A^T^ and QLb11B possessed the complete indole‐3‐acetamide pathway, which converts tryptophan into indole‐3‐acetamide and subsequently into IAA, by the activity of an tryptophan 2‐monooxygenase and an amidase, respectively (McClerklin et al. 2018), (Figure S4 and Table S12). The other strains only had incomplete pathways (Figure S4 and Table S12).

To determine if these strains could indeed synthesise IAA, we estimated the production of indole, as an indirect measurement (Spaepen et al. 2007; McClerklin et al. 2018) using the API 20 NE kit. None of the nodule‐isolated *Pseudomonas* strains exhibited indole production (Table S10).

In addition, we tested the ability of the isolates to solubilise phosphate and produce iron chelators. In general, all isolates belonging to the same species exhibited similar phenotypes. *P. marginalis* and *P. alvendae* isolates were the strongest siderophore producers, while *P. zarinae* and *P. alvendae* were the best phosphate solubilisers. *P. monachiensis* isolates were weak in both activities (Figure S5).

### 3.5 Nodule‐Isolated *Pseudomonas* Promote Shoot Growth and Induce Earlier Root Hair Proliferation in *L. burttii* but Do Not Affect the Formation of Nodules

**FIGURE 4:**
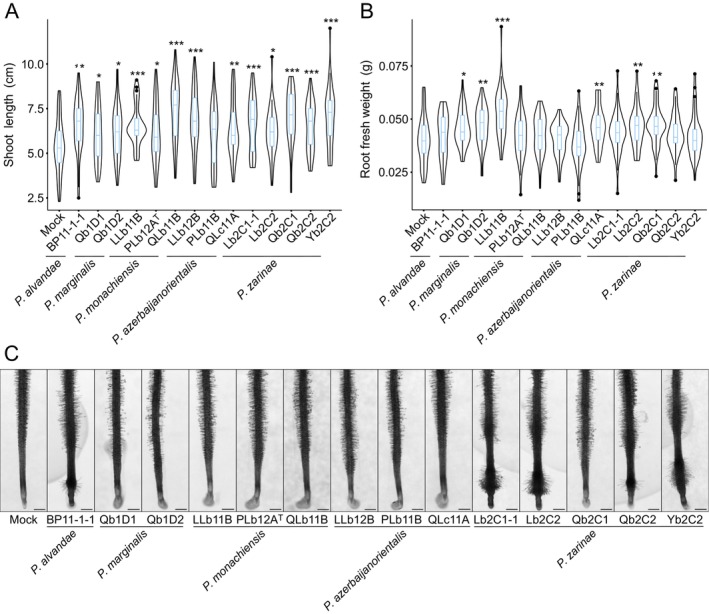
Nodule‐isolated *Pseudomonas* promote *Lotus burttii* growth. Shoot length (A) and root fresh weight (B) phenotypes of plants inoculated with nodule‐isolated *Pseudomonas* strains under axenic conditions. Seedlings of *L. burttii* were grown in sand:vermiculite mixtures supplemented with FAB medium containing 5 mM KNO_3_ (FAB_5mM_) and individually inoculated with *Pseudomonas* strains (OD_600_: 0.005). Mock plants were treated with FAB_5mM_. All plants were incubated under a long‐day photoperiod at 24°C for 4 weeks. Two independent experiments were conducted each comprising 20 plants per treatment. Student's *t*‐tests were conducted using R version 4.2.2 (R Core Team, 2013). * (*p* < 0.05), ** (*p* < 0.01), and *** (*p* < 0.001). (C) Root hair phenotype. *L. burttii* seedlings were transplanted onto fresh 0.5x B5 agar medium, and root tips were spot inoculated with 20 μL of *Pseudomonas* suspensions (OD_600_: 0.005), or mock‐treated with 20 μL of sterile water. Plates were then incubated under a long‐day photoperiod at 24°C for 5 days. Two independent experiments were conducted each comprising 20 plants per treatment. A representative plant from each treatment is shown. Scale bar: 1 mm.

To assess the ability of nodule‐isolated *Pseudomonas* strains in promoting plant growth, we inoculated *L. burttii* seedlings with *Pseudomonas* under axenic conditions. Already, at 1 week post inoculation, enhancement in shoot growth was observed compared to the control treatment. After four‐weeks of inoculation, 13 nodule‐isolated *Pseudomonas* strains significantly promoted shoot growth of *L. burttii* (Figure 4A). Plants inoculated with isolate PLb11B, were slightly bigger than mock treated plants, but the difference was not significant in all repetitions. Additionally, strains Qb2C2 and Yb2C2 significantly increased *L. burttii* root length (Figure S6), while six other strains significantly enhanced root fresh weight (Figure 4B).

The proliferation of root hairs significantly enhances the total surface area of roots and facilitates nutrient uptake (Cui et al. 2018). To investigate if *Pseudomonas* isolates can alter root hair development, we grew *L. burttii* seedlings on plates and inoculated them with the different isolates under axenic conditions. Among the 14 nodule‐isolated *Pseudomonas*, five isolates (BP11‐1‐1, Lb2C1–1, Lb2C2, Qb2C2 and Yb2C2) induced an early onset of root hair proliferation compared to mock treatment (Figure 4C).

To determine whether the nodule isolates influence root nodule development, we co‐inoculated *L. burttii* seedlings with *Pseudomonas* strains and the compatible symbiont *Mesorhizobium* sp. DC‐1.5, previously isolated from *L. burttii* (Crosbie et al. 2022). Co‐inoculation with *Pseudomonas* did not significantly affect the number of nodules formed (Figure S7).

## 4 Discussion

### 4.1 Nodule‐Isolated *Pseudomonas* Are Phylogenetically and Genomically Diverse

Root nodules are protected from the surroundings by a suberised cell layer, forming a barrier that likely restricts the range of bacteria able to colonise them (Venado et al. 2022). Consequently, nodules are typically colonised by a narrow range of bacteria, which often includes *Pseudomonas* (Hnini and Aurag 2024; Yu, Crosbie, and Marín Arancibia 2025). Most studies documenting the presence of *Pseudomonas* in root nodules, rely in either 16S rRNA gene meta‐barcoding (Lu et al. 2017; Sharaf et al. 2019; Han et al. 2020; Mayhood and Mirza 2021) or culture‐based methods followed up by 16S rRNA gene sequencing (Aserse et al. 2013; Tapia‐García et al. 2020; Flores‐Duarte et al. 2022). This gives a very narrow view of the genetic diversity of *Pseudomonas* able to colonise nodules, as the 16S rRNA gene in *Pseudomonas* is highly conserved, making it unsuitable for precise taxonomic classification at the species level (Gomila et al. 2015).

In this study, 14 *Pseudomonas* strains were isolated from the root nodules of *L. burttii* and *L. corniculatus*. Thirteen isolates originated from *L. burttii*, while none were obtained from *L. japonicus* . *L. burttii* is known for its promiscuity in nodulating with a wide variety of rhizobia species, whereas *L. japonicus* exhibits a much more restrictive nodulation profile (Zarrabian et al. 2022). Whole genome sequencing was conducted to elucidate the taxonomic classification of the *Pseudomonas* isolates. Genomes varied in size from 6.2 to 7.0 Mbp and GC content ranged from 60.03% to 60.92% (Table S3). Phylogenetic, ANI and dDDH analyses identified five species in three different subgroups within the *P. fluorescens* group, with *P. monachiensis* being a novel bacterial species (Figures 1 and S2, and Tables S5–S8). Among these five species, strains belonging to *P. marginalis* ( *P. fluorescens* subgroup) have been identified as soft rot phytopathogens (Liao and Wells 1987; Janse et al. 1992). The type strains of *P. alvandae* and *P. zarinae* ( *P. corrugata* subgroup) as well as *P. azerbaijanorientalis* ( *P. jessenii* subgroup), were all isolated from the rhizosphere of wheat plants, but their interactions with host plants have not been studied (Girard et al. 2021). Our results support a plant‐associated lifestyle for members of these species.

The finding that all strains isolated in this work belong to the *P. fluorescens* group is consistent with previous findings involving other *Pseudomonas* strains isolated from *Lotus parviflorus* nodules. For instance, *Pseudomonas helmanticensis* LpB5b, *Pseudomonas koreensis* LpB16d, and *P. fluorescens* strain LpB12a, were isolated from *Lotus* and assigned via 16S rRNA gene phylogeny to the *P. koreensis* and *P. fluorescens* subgroups, respectively (Soares et al. 2020). *P. helmanticensis* LpA6 was identified as *P. koreensis* ( *P. koreensis* subgroup) based on the 16S rRNA and *atpD* genes and as *P. jessenii* ( *P. jessenii* subgroup) based on the *recA* gene (Soares et al. 2020). While *Pseudomonas* isolated from *G. max* belong to a wider range of groups including the *P. fluorescens* (e.g., *Pseudomonas* sp. 108 ia, 108 ic, 115 ic, 140 ic, NT 76 ie and Sneb1997), *Pseudomonas aeruginosa* (e.g., *Pseudomonas* sp. LSE‐2), *Pseudomonas putida* (e.g., *Pseudomonas* sp. 134 ia and DD201) and *Pseudomonas stutzeri* (e.g., *Pseudomonas* sp. 138 id) groups (Zhao et al. 2018, 2021; Kumawat et al. 2019; Tokgöz et al. 2020). The recurring isolation of members of specific groups in different hosts suggests a degree of specificity in the interaction and raises the question if the same genetic programs controlling selectivity/promiscuity with rhizobia control this process (Zarrabian et al. 2022).

### 4.2 Plant Growth‐Promoting Potential by Nodule‐Isolated *Pseudomonas*

*Pseudomonas* have been frequently reported to promote plant health by alleviating abiotic and biotic stresses and promoting overall plant growth through different mechanisms (Santoyo et al. 2012). This is also true for *Pseudomonas* isolated from nodules of various leguminous plants (Martínez‐Hidalgo and Hirsch 2017; Hnini and Aurag 2024; Yu, Crosbie, and Marín Arancibia 2025). Here, we assessed the PGP abilities in silico, in vitro and in planta of the 14 *Pseudomonas* strains isolated from *Lotus* nodules. Our findings revealed that the majority of nodule‐isolated *Pseudomonas* strains significantly enhanced shoot growth in *L. burttii*, with some strains also exhibiting increased root length and fresh weight. In previous studies, Gopalakrishnan et al. reported that *Pseudomonas* sp. IC‐76, isolated from nodules of *Cicer arietinum* , promoted shoot and root biomass (Gopalakrishnan et al. 2015). Similarly, *Pseudomonas* strains 108 ia, 113 id, 115 ic, 131 id, 134 ia, 138 id, 140 ic, NT 76 ia and NT 76 ie, isolated from *G. max* nodules, enhanced shoot height and biomass (Tokgöz et al. 2020), while strains N4 and N8 isolated from wild *Medicago* species nodules increased seed germination, nodule numbers, and root and shoot biomass (Flores‐Duarte et al. 2022).

In addition, we observed that five strains belonging to *P. alvendae* and *P. zarinae* promoted the early onset of root hair formation (Figure 4C). Root hair formation and elongation is modulated by IAA (Pitts et al. 1998) and *Pseudomonas* have been shown to produce this hormone (Noreen et al. 2012; Bharucha et al. 2013; Bakaeva et al. 2020). Our functional prediction of PGPT revealed an abundance of IAA‐related genes in the genomes of the nodule‐isolated *Pseudomonas* strains, suggesting a potential mechanism underlying their ability to induce early onset of root hair proliferation. However, after mapping the prediction results from PLaBAse and comparing them to previous references, we found that out of the 14 nodule‐isolated *Pseudomonas* strains, only one *P. alvendae* and three *P. monachiensis* possessed a complete IAA biosynthetic pathway via the IAM pathway, utilising tryptophan 2‐monooxygenase and amidase (Spaepen et al. 2007; McClerklin et al. 2018). The other strains had only incomplete pathways. Moreover, none of the strains produced indole under the tested conditions (Table S10), which is a rough estimation of IAA. Therefore, there was no correlation between IAA production in vitro and the observed root hair phenotype, suggesting that another mechanism is likely responsible for root hair promotion. For instance, ethylene stimulates root hair development (Li et al. 2022), while phosphate starvation promotes their elongation (Bhosale et al. 2018). The metabolic activity of *Pseudomonas* could alter the local pH, thereby affecting phosphate solubility (Miller et al. 2010; Oteino et al. 2015; Kumawat et al. 2019; Wasule et al. 2023), and/or specific strains could trigger endogenous ethylene synthesis in the host (Singh et al. 2015).

The observed changes in root hair formation are intriguing when considered in the context of the symbiotic interaction with rhizobia. Rhizobia colonise the host root through tubular structures known as infection threads (de Carvalho‐Niebel et al. 2024), which develop in a highly controlled manner within the so‐called susceptibility zone. Alterations in root hair development could potentially impact the root's susceptibility to rhizobia, thereby influencing the efficiency of infection. Nevertheless, co‐inoculation of *Pseudomonas* did not significantly affect the number of root nodules formed in response to *Mesorhizobium*. In the future, it will be important to investigate how these nodule‐isolated *Pseudomonas* strains affect nodule function. This research is particularly relevant in the context of mixed inoculants containing non‐rhizobiales bacteria, which could offer different benefits to the plant but might also influence the interaction between rhizobia and the host.

## 5 Conclusion

The *Pseudomonas* isolated from *Lotus* nodules in this study belong to the *P. fluorescens* group and are genetically diverse. These strains lack known symbiotic genes but have the capacity to significantly enhance shoot growth and, in some instances, stimulate early onset of root hair development. This correlates with an abundance of in silico PGPTs. In addition, several strains can solubilise phosphate and/or secrete siderophores, while not interfering with symbiotic nodule formation. These results highlight the applied potential of nodule‐isolated *Pseudomonas* for the formulation of mixed inoculants together with nitrogen‐fixing rhizobia.

## Author Contributions

**Yu‐Hsiang Yu:** conceptualization, formal analysis, visualization, writing – original draft, methodology, investigation, writing – review and editing, data curation. **Julian Kurtenbach:** methodology, investigation, writing – review and editing. **Duncan Crosbie:** methodology, investigation, writing – review and editing. **Andreas Brachmann:** methodology, investigation, writing – review and editing. **Macarena Marín Arancibia:** conceptualization, writing – original draft, writing – review and editing, project administration, supervision, funding acquisition.

## Conflicts of Interest

The authors declare no conflicts of interest.

## Supporting information


Data S1.

Data S2.

## Data Availability

The BioProject/BioSample/Accession numbers of the assembled genomes are listed in Table S3. Sanger sequencing data of the 16S rRNA sequences for the new species have been uploaded to NCBI with the following accession numbers: PP968549 (PLb12A^T^), PP968550 (LLb11B), and PP968551 (QLb11B).

## Appendix A

### Description of *Pseudomonas monachiensis* sp. nov

*Pseudomonas monachiensis* (mon.a.chi.en'sis; N.L. gen. n. monachiensis of Monachium, Munich, Germany, where the type strain was isolated).

Bacteria grow on LB and 20Q medium. Colonies develop within 1 day in LB medium, and are circular, convex, smooth, opaque, and beige to slightly yellowish in colour with 1.7–2.2 mm diameter at 28°C. Growth is observed at temperatures between 4°C and 28°C and weak growth at 37°C. The major fatty acids (> 5%) are C_10:0 3‐OH_, C_12:0 3‐OH_, C_12:1 3‐OH ω7c_, C_16:0_, C_16:1 ω7c_, and C_17:0 cyclo ω7c_. The genome size of the type strain (PLb12A^T^) is 6.23 Mbp with a GC content of 60.04%.

The type strain PLb12A^T^ (DSM 116615, CCOS 2100, LMG 33494) was isolated in 2019 from a root nodule of *Lotus burttii* in Munich, Germany. The BioProject, BioSample, and Accession number of the assembled genome are PRJNA1195397, SAMN45219950, and JBJVNW000000000, respectively.
