# Supplementary material for: Pseudomonas Species Isolated From Lotus Nodules Are Genetically Diverse and Promote Plant Growth
Source: Environ Microbiol. 2025 Feb 27;27(3):e70066. doi: 10.1111/1462-2920.70066 (PMC11868405; doi:10.1111/1462-2920.70066)
Supplement: Supplementary file 2 — Data S2. [file EMI-27-e70066-s001.docx]

**Supporting information**

**TABLE S1** Strains used in this study.

**TABLE S2** Strains used in the phylogenetic and the plant growth promoting traits annotation analyses.

**TABLE S3** Genome information of nodule-isolated *Pseudomonas*.

**TABLE S4** Genes used for the construction of the core genome phylogenomic tree.

**TABLE S5** FastANI analysis of nodule-isolated *Pseudomonas* strains with closely related type strains.

**TABLE S6** OrthoANI analysis of *Pseudomonas* strains LLb11B, PLb12A^T^, and QLb11B with closely related type strains.

**TABLE S7** Digital DNA-DNA hybridisation analysis of *Pseudomonas* strains LLb11B, PLb12A^T^, and QLb11B with closely related type strains.

**TABLE S8** Species assignment of *Pseudomonas* strain PLb12A^T^ by the GTDB-Tk database.

**TABLE S9** Phenotypic characterisation of *Pseudomonas monachiensis* sp. nov.

**TABLE S10** API^®^ 20 NE assay results.

**TABLE S11** Functional plant growth promoting trait counts predicted by the PGPT-Pred tool on the PLaBAse server.

**TABLE S12** Indole-3-acetic acid biosynthetic pathways and gene distribution in nodule-isolated *Pseudomonas* and their closest type strains.

**FIGURE S1** 16S rRNA gene phylogenetic tree of nodule-isolated *Pseudomonas*.

**FIGURE S2** Core genome phylogenomic tree of nodule-isolated *Pseudomonas*.

**FIGURE S3** Colony morphology of *Pseudomonas monachiensis* sp. nov. PLb12A^T^.

**FIGURE S4** Putative indole-3-acetic acid biosynthetic pathways of nodule-isolated *Pseudomonas* strains and their closest type strains.

**FIGURE S5** Nodule-isolated *Pseudomonas* strains produce siderophores and solubilise phosphate *in vitro*.

**FIGURE S6** Nodule-isolated *Pseudomonas* promote root growth in *Lotus burttii*.

**FIGURE S7** Nodule-isolated *Pseudomonas* strains do not increase nodule numbers during co-inoculation.

**
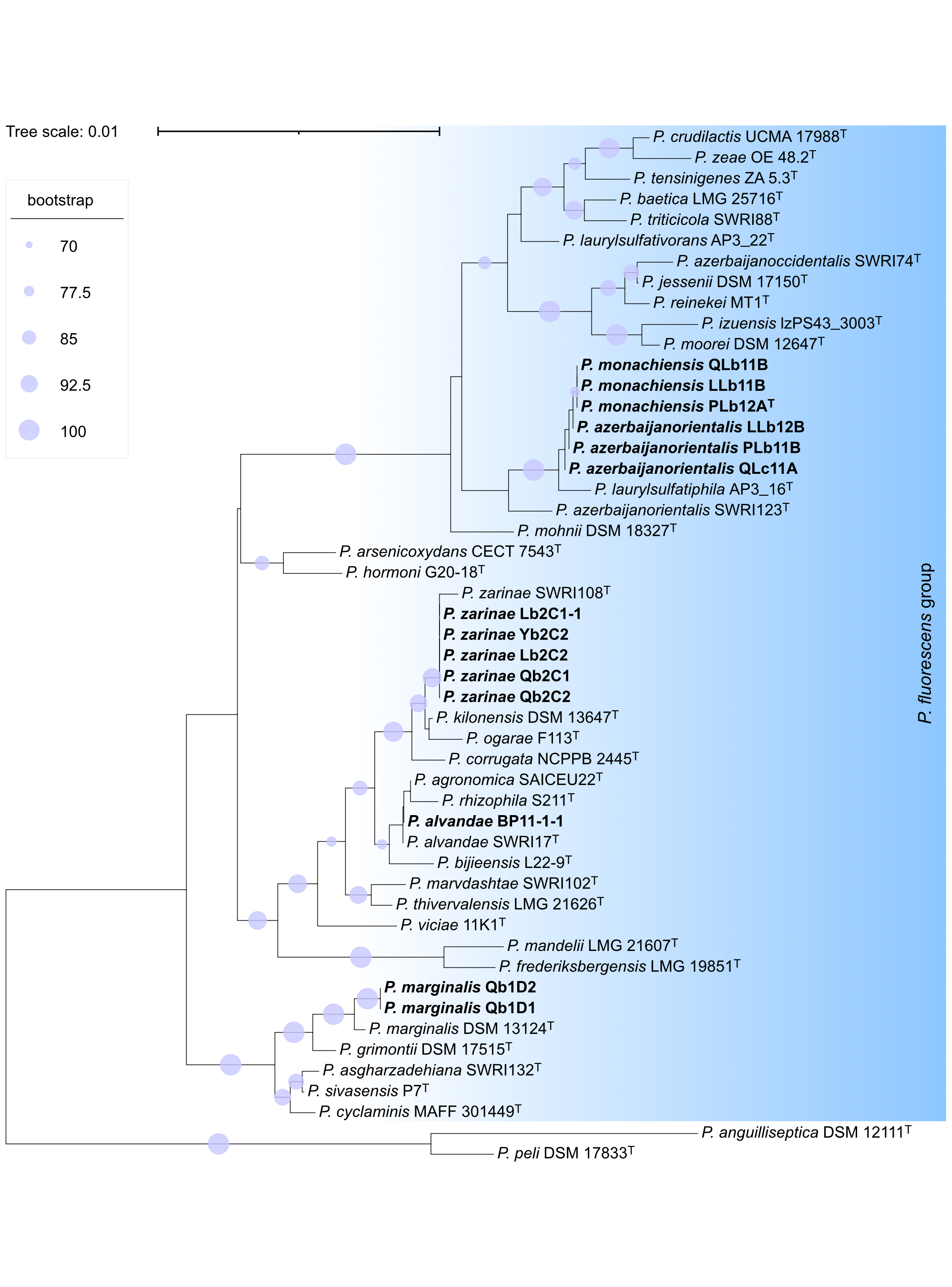
**

**FIGURE S1** 16S rRNA gene phylogenetic tree of nodule-isolated *Pseudomonas*. The phylogenetic tree was constructed using the 16S rRNA sequences of 14 nodule-isolated *Pseudomonas* and 36 *Pseudomonas* type strains in the TYGS server (Meier-Kolthoff and Göker, 2019). Two groups were included in the tree: *Pseudomonas fluorescens* group and the *Pseudomonas anguilliseptica* group. The type strains *P. anguilliseptica* DSM 12111^T^ and *Pseudomonas peli* DSM 17833^T^ in the *P. anguilliseptica* group were used as outgroup. Nodule isolates are highlighted in bold. Branches are annotated with pseudo-bootstrap support values (>70%) from 100 replications, averaging at 69.8% branch support.

**
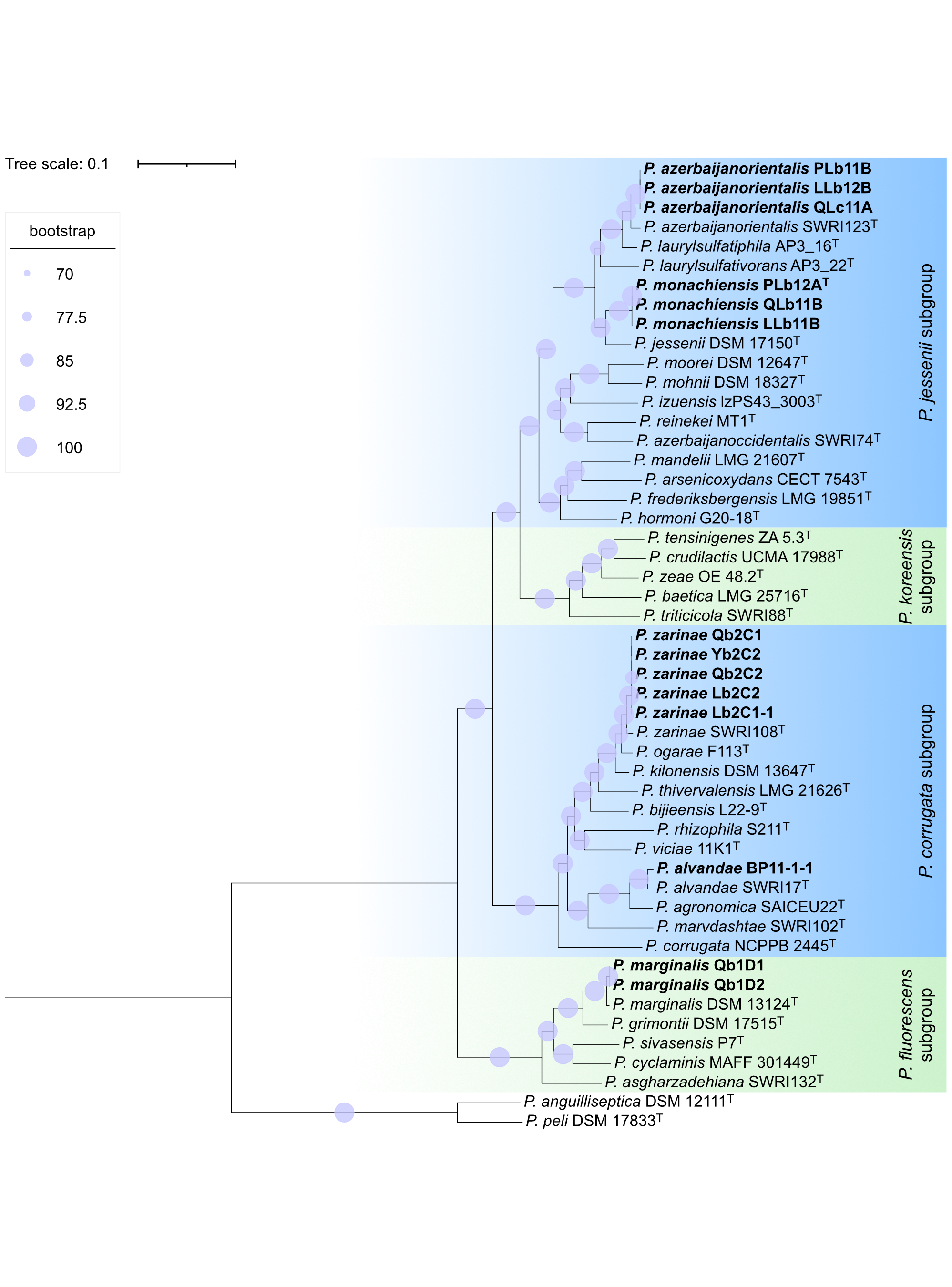
**

**FIGURE S2** Core genome phylogenomic tree of nodule-isolated *Pseudomonas*. The phylogenomic tree was constructed using the 1,423 core genes of 14 nodule-isolated *Pseudomonas* and 36 *Pseudomonas* type strains in RAxML-HPC2 (Stamatakis, 2014). Two groups were included in the tree: *Pseudomonas fluorescens* group (including *Pseudomonas jessenii*, *Pseudomonas koreensis*, *Pseudomonas corrugata*, and *P. fluorescens* subgroups) and the *Pseudomonas anguilliseptica* group. The type strains *P. anguilliseptica* DSM 12111^T^ and *Pseudomonas peli* DSM 17833^T^ in the *P. anguilliseptica* group were used as outgroup. Nodule isolates are highlighted in bold. Bootstrap support values greater than 70%, based on 1,000 replications, are indicated under the branches.

**
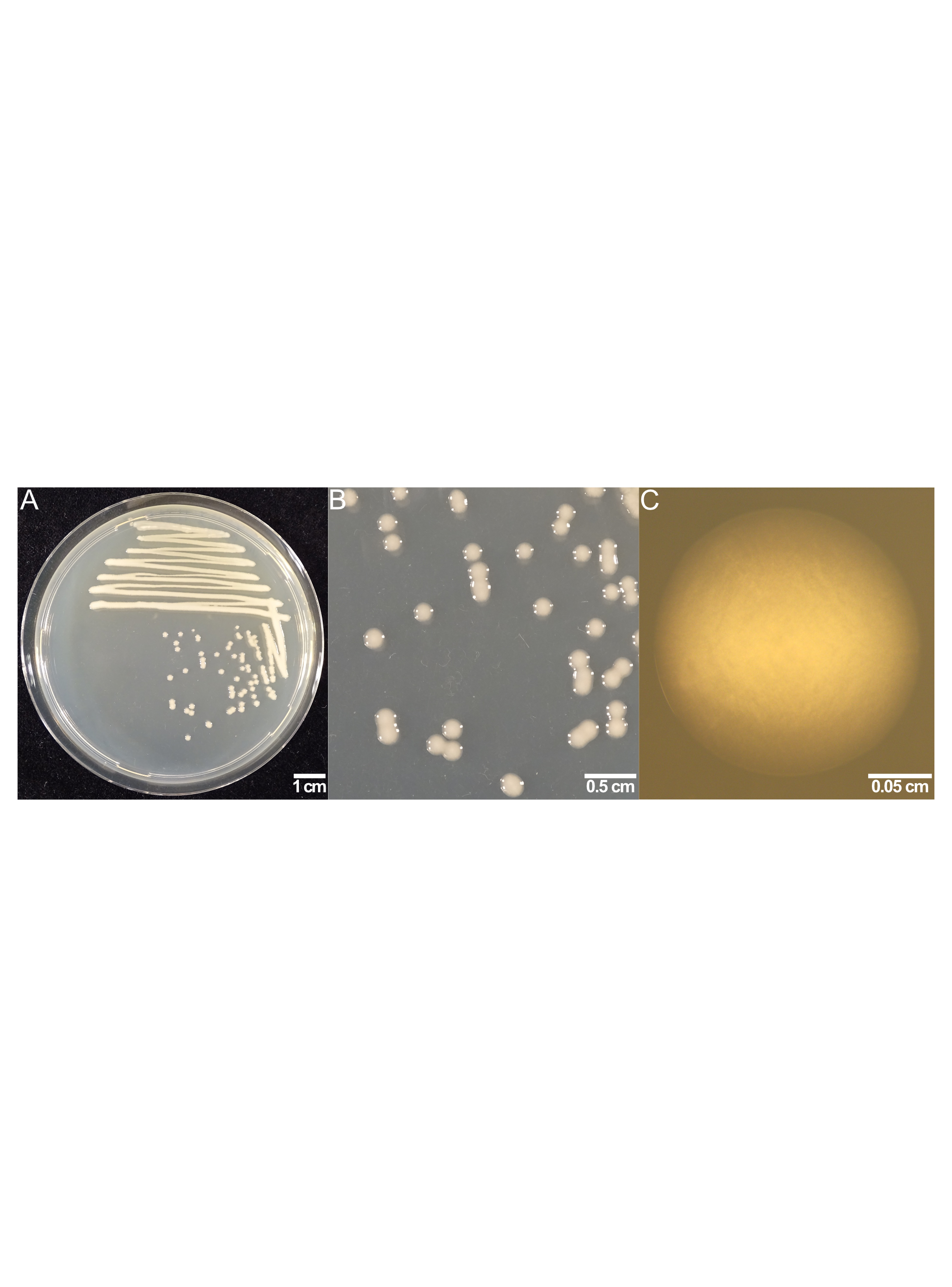
**

**FIGURE S3** Colony morphology of *Pseudomonas monachiensis* sp. nov. PLb12A^T^. (A) plate overview of streaked PLb12A^T^ with single colonies after 24 h of incubation on LB agar medium at 28°C. (B and C) Close-up of single colonies.


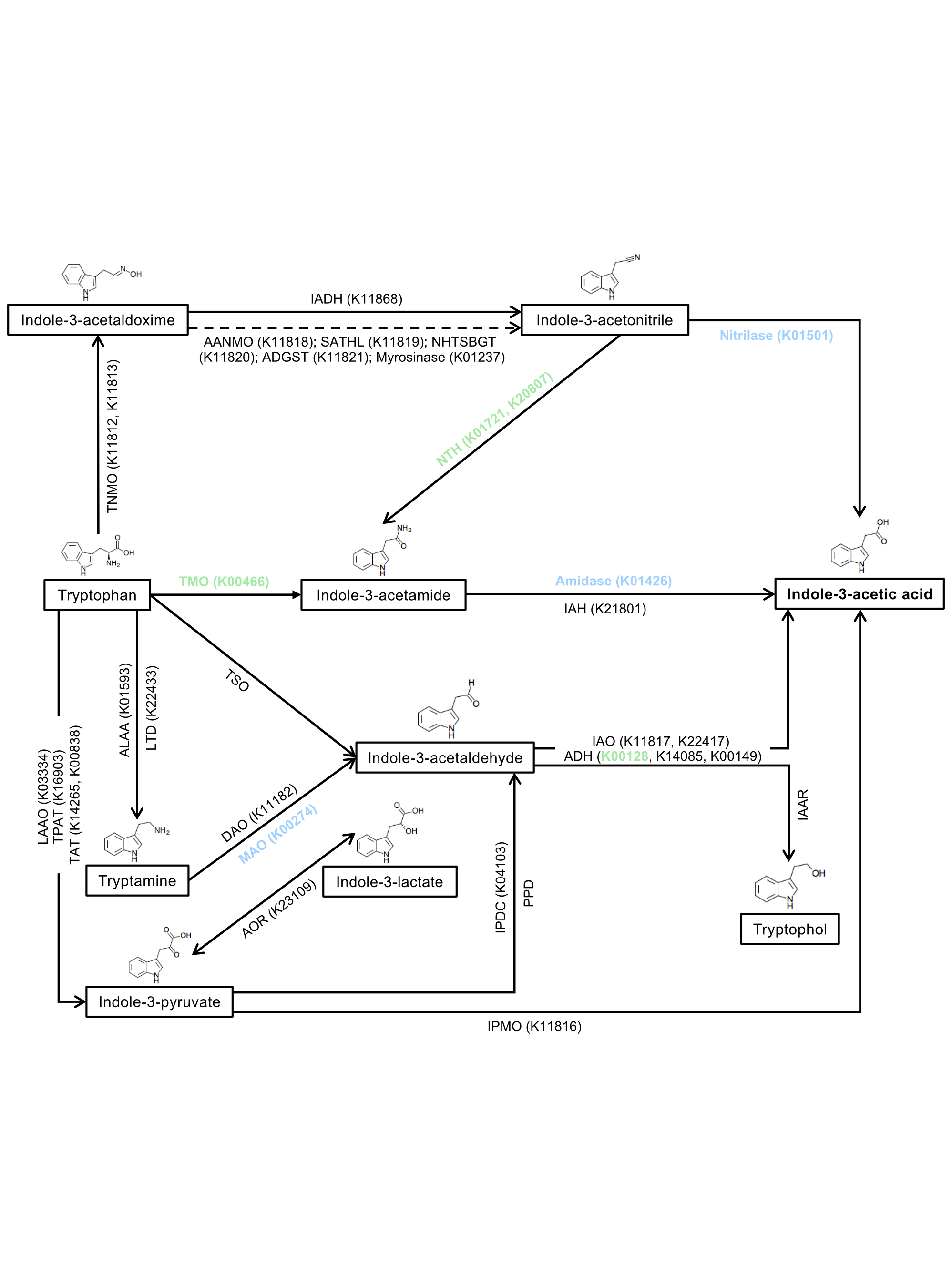
 **FIGURE S4** Putative indole-3-acetic acid biosynthetic pathways of nodule-isolated *Pseudomonas* strains and their closest type strains. Putative indole-3-acetic acid (in bold black) biosynthetic pathways were predicted based on the PLaBAse server (Patz et al., 2021) and the KEGG database (Kanehisa and Goto, 2000). Enzymes highlighted in bold blue are present in all strains, while those highlighted in bold green are present only in specific strains. This figure was modified from (Spaepen et al., 2007; McClerklin et al., 2018), and the chemical compound images were sourced from the KEGG database (Kanehisa and Goto, 2000). The dashed line indicates that intermediate products in the biosynthetic pathways are not shown. AANMO: aromatic aldoxime N-monooxygenase; ADGST: aromatic desulfoglucosinolate sulfotransferase; ADH: aldehyde dehydrogenase (NAD+); ALAA: aromatic-L-amino-acid; AOR: aromatic 2-oxoacid reductase; DAO: diamine oxidase; IAAR: indole-3-acetaldehyde reductase (NAPDH); IADH: indoleacetaldoxime dehydratase; IAH: indoleacetamide hydrolase; IAO: indole-3-acetaldehyde oxidase; IPDC: indolepyruvate decarboxylase; IPMO: indole-3-pyruvate monooxygenase; LAAO: L-amino-acid oxidase; LTD: L-tryptophan decarboxylase; MAO: monoamine oxidase; NHTSBGT: N-hydroxythioamide S-beta-glucosyltransferase; NTH: nitrile hydratase subunit alpha/beta; PPD: phenylpyruvate decarboxylase; SATHL: S-alkyl-thiohydroximate lyase SUR1; TAT: tryptophan aminotransferase; TMO: tryptophan 2-monooxygenase; TNMO: tryptophan N-monooxygenase; TPAT: L-tryptophan-pyruvate aminotransferase; TSO: tryptophan side-chain oxidase.


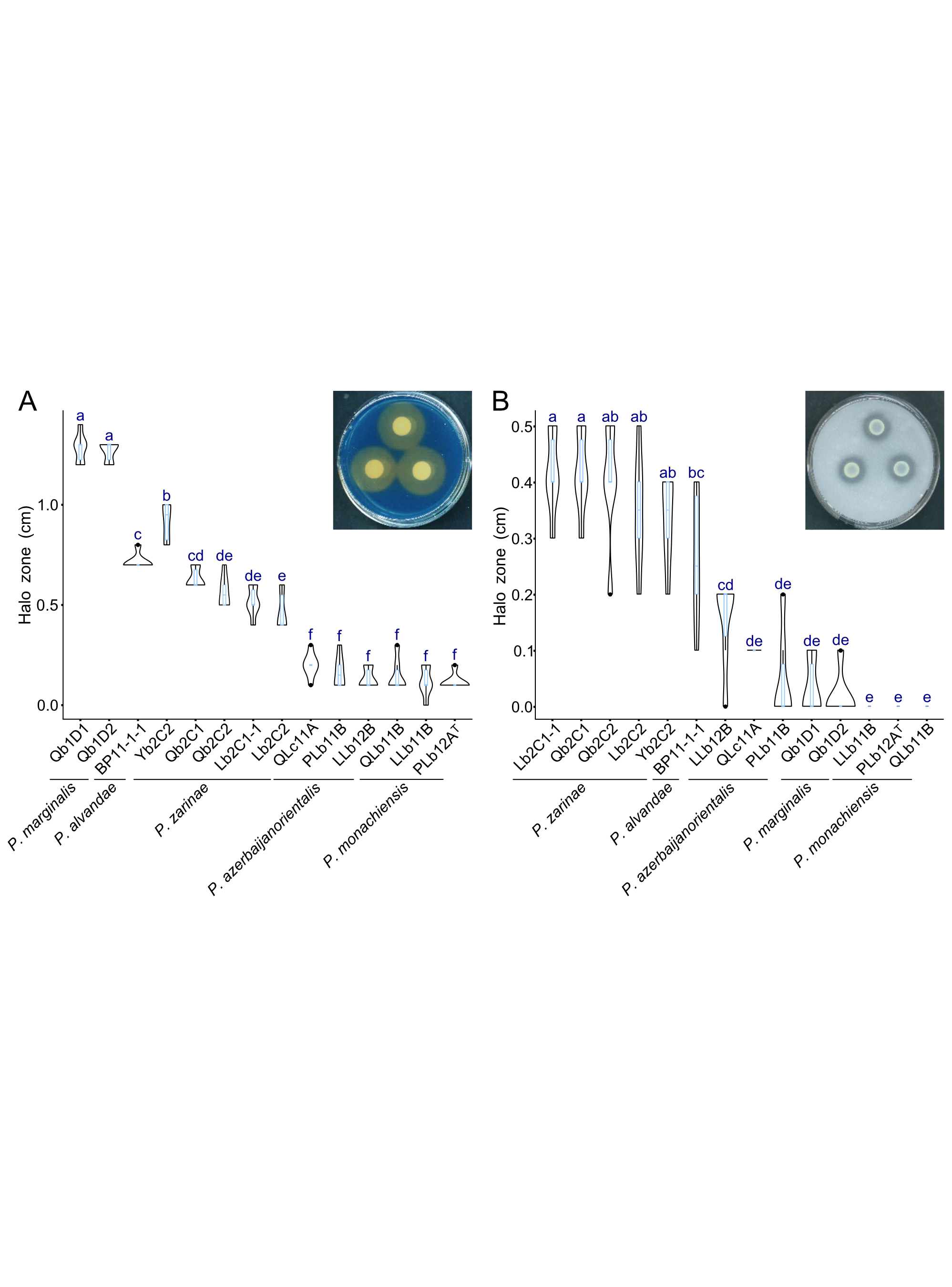


**FIGURE S5** Nodule-isolated *Pseudomonas* strains produce siderophores and solubilise phosphate *in vitro*. The siderophore production assay was performed using Chrome azurol S (CAS) agar (Alexander et al., 1991), and the phosphate solubilisation assay was conducted using Pikovskaya’s agar (Pikovskaya, 1948). Suspensions of nodule-isolated *Pseudomonas* strains were adjusted to an OD_600_ of 1.0 with sterile water, and three replicates of each strain were spotted onto the respective agar plates. Plates were incubated at 28°C, and measurements were taken every 24 hours over 7 days to monitor growth and the formation of halo zones. Two independent experiments were performed for each assay. One-way ANOVA followed by Tukey’s HSD test was performed to evaluate differences among strains using R version 4.2.2 (R Core Team, 2013).


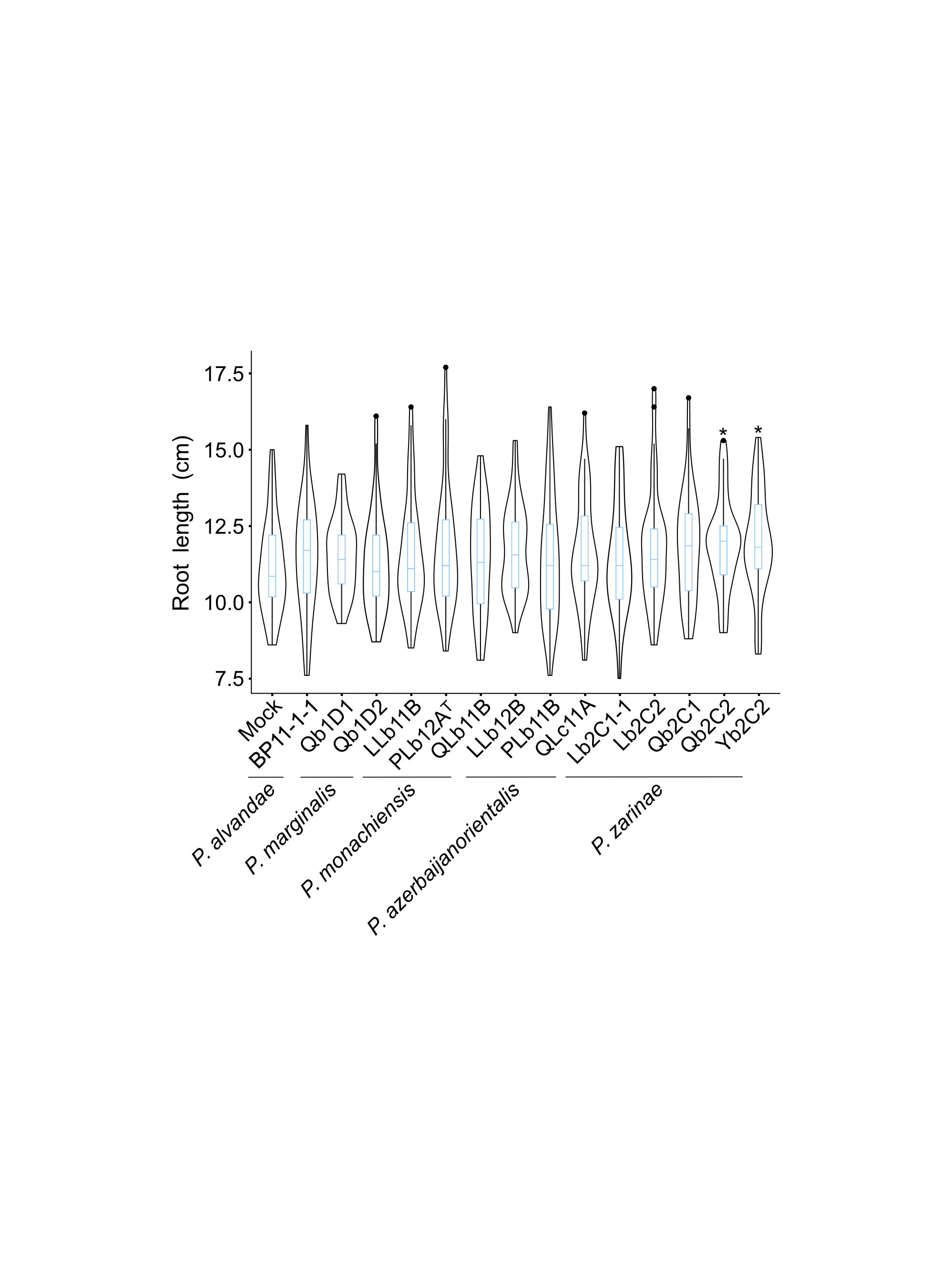


**FIGURE S6** Nodule-isolated *Pseudomonas* promote root growth in *Lotus burttii*. Root length phenotype of plants inoculated with nodule-isolated *Pseudomonas* strains under axenic conditions. Seedlings of *L. burttii* were grown in sand:vermiculite mixtures supplemented with FAB medium containing 5 mM KNO_3_ (FAB_5mM_) and individually inoculated with *Pseudomonas* strains (OD_600_ : 0.005). Mock plants were treated with FAB_5mM_. All plants were incubated under a long-day photoperiod at 24°C for four weeks. Two independent experiments were conducted each comprising 20 plants per treatment. Student's *t*-tests were conducted using R version 4.2.2 (R Core Team, 2013). * indicates *P* < 0.05.

**
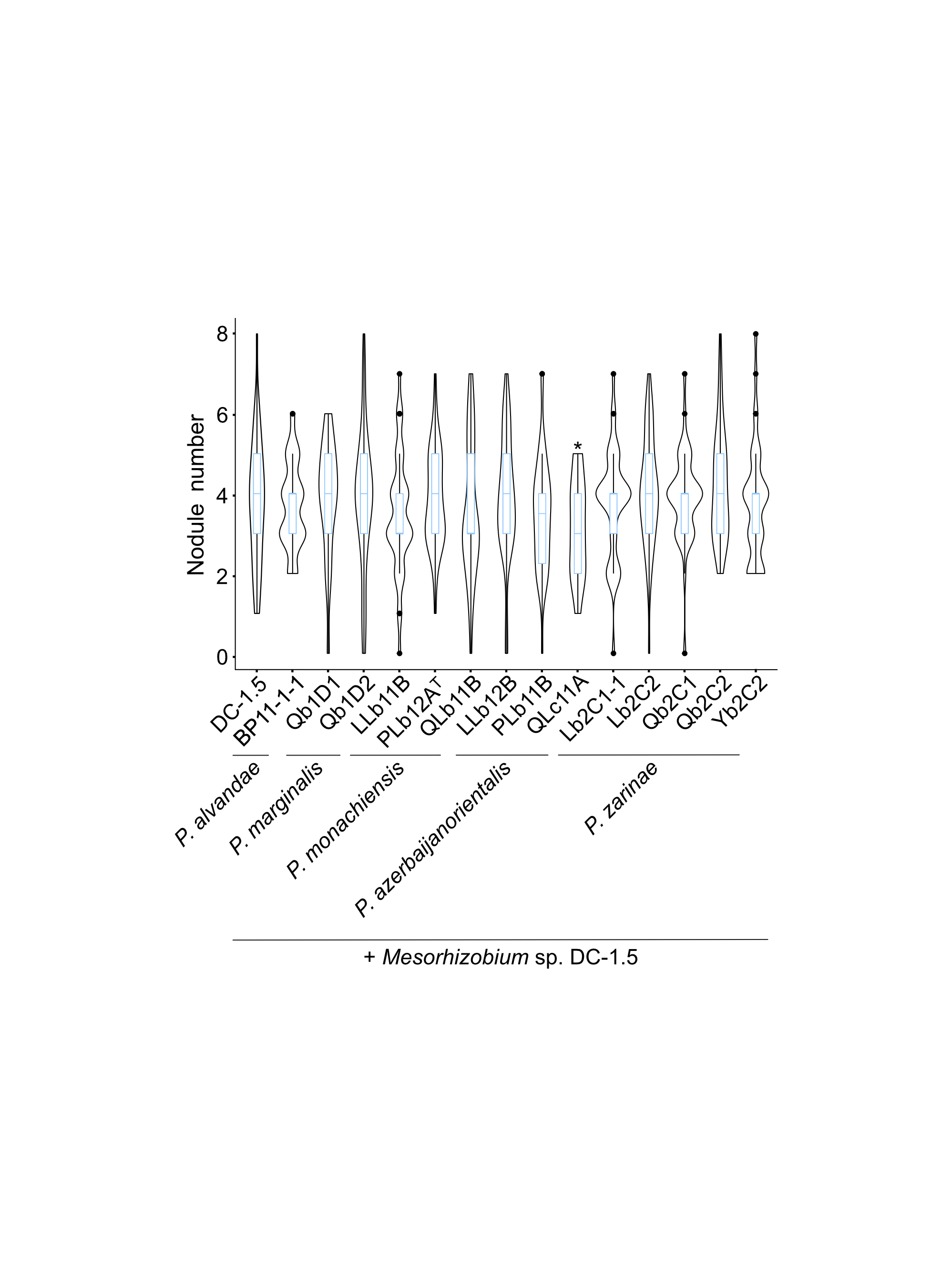
**

**Figure S7** Nodule-isolated *Pseudomonas* strains do not increase nodule numbers during co-inoculation. Seedlings were grown in sand:vermiculite mixtures supplemented with FAB medium containing 0.1 mM KNO_3_ (FAB_0.1mM_). *Pseudomonas* and *Mesorhizobium* sp. DC-1.5 suspensions were diluted to an OD_600_ of 0.01 using FAB_0.1mM_ and mixed in a 1:1 ratio to achieve a final OD_600_ of 0.005 for each strain. One milliliter of this mixture was inoculated per seedling. Single inoculation with *Mesorhizobium* sp. DC-1.5 was used as the control. Three weeks post-inoculation, plants were harvested, and nodule numbers were counted. Two independent experiments were performed, each comprising 20 plants per treatment. Student’s *t*-tests were conducted using R version 4.2.2 (R Core Team, 2013). * indicates *P* < 0.05.

**REFERENCES**

Adeniji, A.A., Aremu, O.S., Loots, D.T., and Babalola, O.O. (2020) *Pseudomonas fulva* HARBPS9.1: candidate anti-*Fusarium* agent in South Africa. *European Journal of Plant Pathology* 157: 767-781.

Afordoanyi, D.M., Diabankana, R.G.C., Miftakhov, A.K., Kuchaev, E.S., and Validov, S.Z. (2022) Genomic features of *Pseudomonas putida* PCL1760: a biocontrol agent acting via competition for nutrient and niche. *Applied Microbiology* 2: 749-765.

Alexander, D.B., and Zuberer, D.A. (1991) Use of chrome azurol S reagents to evaluate siderophore production by rhizosphere bacteria. *Biology and Fertility of soils*, 12: 39-45.

Berendsen, R.L., van Verk, M.C., Stringlis, I.A., Zamioudis, C., Tommassen, J., Pieterse, C.M., and Bakker, P.A. (2015) Unearthing the genomes of plant-beneficial *Pseudomonas* model strains WCS358, WCS374 and WCS417. *BMC Genomics* 16: 539.

Calderón, C.E., Ramos, C., de Vicente, A., and Cazorla, F.M. (2015) Comparative genomic analysis of *Pseudomonas chlororaphis* PCL1606 reveals new insight into antifungal compounds involved in biocontrol. *Molecular Plant-Microbe Interactions* 28: 249-260.

Crosbie, D.B., Mahmoudi, M., Radl, V., Brachmann, A., Schloter, M., Kemen, E., and Marín, M. (2022) Microbiome profiling reveals that *Pseudomonas* antagonises parasitic nodule colonisation of cheater rhizobia in *Lotus*. *New Phytologist* 234: 242-255.

Furmanczyk, E.M., Kaminski, M.A., Lipinski, L., Dziembowski, A., and Sobczak, A. (2018) *Pseudomonas laurylsulfatovorans* sp. nov., sodium dodecyl sulfate degrading bacteria, isolated from the peaty soil of a wastewater treatment plant. *Systematic and Applied Microbiology* 41: 348-354.

Garrido-Sanz, D., Redondo-Nieto, M., Martin, M., and Rivilla, R. (2021) Comparative genomics of the *Pseudomonas corrugata* subgroup reveals high species diversity and allows the description of *Pseudomonas ogarae* sp. nov. *Microbial Genomics* 7: 000593.

Girard, L., Lood, C., Höfte, M., Vandamme, P., Rokni-Zadeh, H., van Noort, V. et al. (2021) The ever-expanding *Pseudomonas* genus: description of 43 new species and partition of the *Pseudomonas putida* group. *Microorganisms* 9: 1766.

Gu, Y., Wang, J., Xia, Z., and Wei, H.-L. (2020) Characterization of a versatile plant growth-promoting rhizobacterium *Pseudomonas mediterranea* strain S58. *Microorganisms* 8: 334.

Hassen, W., Neifar, M., Cherif, H., Najjari, A., Chouchane, H., Driouich, R.C. et al. (2018) *Pseudomonas rhizophila* S211, a new plant growth-promoting rhizobacterium with potential in pesticide-bioremediation. *Frontiers in Microbiology* 9: 34.

Hauth, F., Buck, H., and Hartig, J.S. (2022) *Pseudomonas canavaninivorans* sp. nov., isolated from bean rhizosphere. *International Journal of Systematic and Evolutionary Microbiology* 72: 005203.

Hernández-Salmerón, J.E., Hernández-León, R., Orozco-Mosqueda, M.D.C., Valencia-Cantero, E., Moreno-Hagelsieb, G., and Santoyo, G. (2016) Draft genome sequence of the biocontrol and plant growth-promoting rhizobacterium *Pseudomonas fluorescens* strain UM270. *Standards in Genomic Sciences* 11: 5.

Jousset, A., Schuldes, J., Keel, C., Maurhofer, M., Daniel, R., Scheu, S., and Thuermer, A. (2014) Full-genome sequence of the plant growth-promoting bacterium *Pseudomonas protegens* CHA0. *Genome Announcements* 2: 10.1128/genomea.00322-00314.

Kanehisa, M., and Goto, S. (2000) KEGG: kyoto encyclopedia of genes and genomes. *Nucleic Acids Research* 28: 27-30.

Krzyżanowska, D.M., Iwanicki, A., Czajkowski, R., and Jafra, S. (2021) High-quality complete genome resource of tomato rhizosphere strain *Pseudomonas donghuensis* P482, a representative of a species with biocontrol activity against plant pathogens. *Molecular Plant-Microbe Interactions* 34: 1450-1454.

Kwak, Y., Park, G.-S., and Shin, J.-H. (2016) High quality draft genome sequence of the type strain of *Pseudomonas lutea* OK2^T^, a phosphate-solubilizing rhizospheric bacterium. *Standards in Genomic Sciences* 11: 51.

Lafi, F.F., Alam, I., Geurts, R., Bisseling, T., Bajic, V.B., Hirt, H., and Saad, M.M. (2016) Draft genome sequence of the phosphate-solubilizing bacterium *Pseudomonas argentinensis* strain SA190 isolated from the desert plant *Indigofera argentea*. *Genome Announcements* 4: 10.1128/genomea.01431-01416.

Liang, J., Wang, S., Yiming, A., Fu, L., Ahmad, I., Chen, G., and Zhu, B. (2021) *Pseudomonas bijieensis* sp. nov., isolated from cornfield soil. *International Journal of Systematic and Evolutionary Microbiology* 71: 004676.

Lin, H., Hu, S., Liu, R., Chen, P., Ge, C., Zhu, B., and Guo, L. (2016) Genome sequence of *Pseudomonas koreensis* CRS05-R5, an antagonistic bacterium isolated from rice paddy field. *Frontiers in Microbiology* 7: 1756.

Liu, Y., Song, Z., Zeng, H., Lu, M., Zhu, W., Wang, X. et al. (2021) *Pseudomonas eucalypticola* sp. nov., a producer of antifungal agents isolated from *Eucalyptus dunnii* leaves. *Scientific Reports* 11: 3006.

McClerklin, S.A., Lee, S.G., Harper, C.P., Nwumeh, R., Jez, J.M., and Kunkel, B.N. (2018) Indole-3-acetaldehyde dehydrogenase-dependent auxin synthesis contributes to virulence of *Pseudomonas syringae* strain DC3000. *PLoS Pathogens* 14: e1006811.

Meier-Kolthoff, J.P., and Göker, M. (2019) TYGS is an automated high-throughput platform for state-of-the-art genome-based taxonomy. *Nature Communications* 10: 2182.

Montes, C., Altimira, F., Canchignia, H., Castro, Á., Sánchez, E., Miccono, M. et al. (2016) A draft genome sequence of *Pseudomonas veronii* R4: a grapevine (*Vitis vinifera* L.) root-associated strain with high biocontrol potential. *Standards in Genomic Sciences* 11: 76.

Nascimento, F.X., Urón, P., Glick, B.R., Giachini, A., and Rossi, M.J. (2021) Genomic analysis of the 1-aminocyclopropane-1-carboxylate deaminase-producing *Pseudomonas thivervalensis* sc5 reveals its multifaceted roles in soil and in beneficial interactions with plants. *Frontiers in Microbiology* 12: 752288.

Nelkner, J., Torres Tejerizo, G., Hassa, J., Lin, T.W., Witte, J., Verwaaijen, B. et al. (2019) Genetic potential of the biocontrol agent *Pseudomonas brassicacearum* (formerly *P. trivialis*) 3Re2-7 unraveled by genome sequencing and mining, comparative genomics and transcriptomics. *Genes (Basel)* 10: 601.

Omoboye, O.O., Geudens, N., Duban, M., Chevalier, M., Flahaut, C., Martins, J.C. et al. (2019) *Pseudomonas* sp. COW3 produces new bananamide-type cyclic lipopeptides with antimicrobial activity against *Pythium myriotylum* and *Pyricularia oryzae*. *Molecules* 24: 4170.

Patz, S., Gautam, A., Becker, M., Ruppel, S., Rodríguez-Palenzuela, P., and Huson, D. (2021) PLaBAse: A comprehensive web resource for analyzing the plant growth-promoting potential of plant-associated bacteria. *Biorxiv* 2021.12: 472471.

Perneel, M., Heyrman, J., Adiobo, A., De Maeyer, K., Raaijmakers, J., De Vos, P., and Höfte, M. (2007) Characterization of CMR5c and CMR12a, novel fluorescent *Pseudomonas* strains from the cocoyam rhizosphere with biocontrol activity. *Journal of Applied Microbiology* 103: 1007-1020.

Pieterse, C.M., Berendsen, R.L., de Jonge, R., Stringlis, I.A., Van Dijken, A.J., Van Pelt, J.A. et al. (2021) *Pseudomonas simiae* WCS417: star track of a model beneficial rhizobacterium. *Plant and Soil* 461: 245-263.

Pikovskaya, R.I. (1948) Mobilization of phosphorus in soil in connection with vital capacity of source microbial species. *Microbiologiya* 17:362-370.

R Core Team, R. (2013). R: A language and environment for statistical computing. URL <https://www.R-project.org/>

Robas Mora, M., Fernández Pastrana, V.M., Oliva, L.L.G., Lobo, A.P., and Jiménez Gómez, P.A. (2023) Plant growth promotion of the forage plant *Lupinus albus* Var. Orden Dorado using *Pseudomonas agronomica* sp. nov. and *Bacillus pretiosus* sp. nov. added over a valorized agricultural biowaste. *Frontiers in Microbiology* 13: 1046201.

Setten, L., Soto, G., Mozzicafreddo, M., Fox, A.R., Lisi, C., Cuccioloni, M. et al. (2013) Engineering *Pseudomonas protegens* Pf-5 for nitrogen fixation and its application to improve plant growth under nitrogen-deficient conditions. *PLoS One* 8: e63666.

Shen, X., Hu, H., Peng, H., Wang, W., and Zhang, X. (2013) Comparative genomic analysis of four representative plant growth-promoting rhizobacteria in *Pseudomonas*. *BMC Genomics* 14: 271.

Sorty, A.M., Zervas, A., García de Salamone, I.E., Nelson, L.M., and Stougaard, P. (2023) *Pseudomonas hormoni* sp. nov., a plant hormone producing bacterium isolated from Arctic grass, Ellesmere Island, Canada. *International Journal of Systematic and Evolutionary Microbiology* 73: 006119.

Spaepen, S., Vanderleyden, J., and Remans, R. (2007) Indole-3-acetic acid in microbial and microorganism-plant signaling. *FEMS Microbiology Reviews* 31: 425-448.

Stamatakis, A. (2014) RAxML version 8: a tool for phylogenetic analysis and post-analysis of large phylogenies. *Bioinformatics* 30: 1312-1313.

Takeuchi, K., Noda, N., and Someya, N. (2014) Complete genome sequence of the biocontrol strain *Pseudomonas protegens* Cab57 discovered in Japan reveals strain-specific diversity of this species. *PLoS One* 9: e93683.

Verhille, S., Baida, N., Dabboussi, F., Izard, D., and Leclerc, H. (1999) Taxonomic study of bacteria isolated from natural mineral waters: proposal of *Pseudomonas jessenii* sp. nov. and *Pseudomonas mandelii* sp. nov. *Systematic and Applied Microbiology* 22: 45-58.

Wang, S., Hu, M., Chen, H., Li, C., Xue, Y., Song, X. et al. (2023) *Pseudomonas forestsoilum* sp. nov. and *P. tohonis* biocontrol bacterial wilt by quenching 3-hydroxypalmitic acid methyl ester. *Frontiers in Plant Science* 14: 1193297.

Wu, L., Xiao, W., Chen, G., Song, D., Khaskheli, M.A., Li, P. et al. (2018) Identification of *Pseudomonas mosselii* BS011 gene clusters required for suppression of rice blast fungus *Magnaporthe oryzae*. *Journal of Biotechnology* 282: 1-9.

Yang, R., Li, S., Li, Y., Yan, Y., Fang, Y., Zou, L., and Chen, G. (2021) Bactericidal effect of *Pseudomonas oryziphila* sp. nov., a novel *Pseudomonas* species against *Xanthomonas oryzae* reduces disease severity of bacterial leaf streak of rice. *Frontiers in Microbiology* 12: 759536.

Zachow, C., Jahanshah, G., de Bruijn, I., Song, C., Ianni, F., Pataj, Z. et al. (2015) The novel lipopeptide poaeamide of the endophyte *Pseudomonas poae* RE* 1-1-14 is involved in pathogen suppression and root colonization. *Molecular Plant-Microbe Interactions* 28: 800-810.

Zengerer, V., Schmid, M., Bieri, M., Müller, D.C., Remus-Emsermann, M.N., Ahrens, C.H., and Pelludat, C. (2018) *Pseudomonas orientalis* F9: a potent antagonist against phytopathogens with phytotoxic effect in the apple flower. *Frontiers in Microbiology* 9: 145.

Zhang, Y., Chen, P., Ye, G., Lin, H., Ren, D., Guo, L. et al. (2019) Complete genome sequence of *Pseudomonas parafulva* PRS09-11288, a biocontrol strain produces the antibiotic phenazine-1-carboxylic acid. *Current Microbiology* 76: 1087-1091.

Zhao, H., Liu, Y.-P., and Zhang, L.-Q. (2019) *In silico* and genetic analyses of cyclic lipopeptide synthetic gene clusters in *Pseudomonas* sp. 11K1. *Frontiers in Microbiology* 10: 544.
